# Microalgae Proteins as Sustainable Ingredients in Novel Foods: Recent Developments and Challenges

**DOI:** 10.3390/foods13050733

**Published:** 2024-02-28

**Authors:** Ornella Kongi Mosibo, Giovanna Ferrentino, Chibuike C. Udenigwe

**Affiliations:** 1 School of Nutrition Sciences, Faculty of Health Sciences, University of Ottawa, Ottawa, ON K1N 9A7, Canada; omosibo@uottawa.ca (O.K.M.); cudenigw@uottawa.ca (C.C.U.); 2 Faculty of Agriculture, Environmental and Food Sciences, Free University of Bozen-Bolzano, Piazza Università 5, 39100 Bolzano, Italy

**Keywords:** microalgae, sustainable proteins, extraction, in vitro digestibility, microalgae-enriched foods

## Abstract

Microalgae are receiving increased attention in the food sector as a sustainable ingredient due to their high protein content and nutritional value. They contain up to 70% proteins with the presence of all 20 essential amino acids, thus fulfilling human dietary requirements. Microalgae are considered sustainable and environmentally friendly compared to traditional protein sources as they require less land and a reduced amount of water for cultivation. Although microalgae’s potential in nutritional quality and functional properties is well documented, no reviews have considered an in-depth analysis of the pros and cons of their addition to foods. The present work discusses recent findings on microalgae with respect to their protein content and nutritional quality, placing a special focus on formulated food products containing microalgae proteins. Several challenges are encountered in the production, processing, and commercialization of foods containing microalgae proteins. Solutions presented in recent studies highlight the future research and directions necessary to provide solutions for consumer acceptability of microalgae proteins and derived products.

## 1. Introduction

Currently, the world population is estimated to be ~8 billion and will increase to ~9.7 billion by 2050. This important increment indicates the need to produce a higher amount of foods, especially meat, and consequently the use of large areas for its production. This approach is considered unsustainable as it implies the utilization of dedicated land, water, nitrogen, and energy sources. In order to meet the urgent protein requirements due to the future insufficient protein supply, alternative protein sources have been recently investigated [1,2].

Proteins are important macronutrients for humans as they are involved in diverse biological processes in the body, such as muscular contraction, tissue repair, or metabolic reactions. Different sources of protein can be used for the human diet. Currently, the major sources are animal- and plant-based. Animal-based protein production depends on an effective supply of plant-based proteins for feed. In turn, plant proteins can be maintained through the expansion and diversification of cultivation areas and the increase in crop yield. This topic evokes controversial debates on land degradation, the loss of biodiversity, and deforestation as the planet increasingly suffers from environmental damages and climate changes caused by the improper use of its resources. This harsh reality underscores the urgent need for sustainable protein sources [2,3].

Moreover, the promotion of veganism by eliminating animal-based foods and consuming sustainable and healthy foods have particularly offered an open door to explore alternative protein sources. In addition to plant-based proteins, other alternative protein sources such as insects, bacteria, and microalgae have been recently investigated. Microalgae offer many advantages, such as their rapid growth, high protein content, a rich amino acid profile, and low risk of pathogens [4]. The term “microalgae” refers to various groups comprising prokaryotic cyanobacteria and eukaryotic photosynthetic microorganisms found within the taxa *Chlorophyta*, *Rhodophyta*, *Glaucocystophyta*, *Euglenophyta*, *Chlorarachniophyta*, *Heterokonta*, *Haptophyta*, *Cryptophyta*, and *Alveolata* [5]. Microalgae are unicellular photosynthetic microorganisms living individually or in colonies. They are mostly eukaryotic even though prokaryotic cyanobacteria are also counted. Cyanobacteria can be unicellular or multicellular. Microalgae can grow in freshwater or saline water. They are photoautotrophs with a high photosynthetic efficiency. They produce biomolecule-rich biomasses with a high protein content and possess cell walls whose composition differs among the different groups. Microalgae cell walls are made up of microfibrils associated with polysaccharides and proteins. For instance, *Chlorella* spp. cell walls are 17–22 nm thick comprising a rigid cell wall (glucosamine and glucose–mannose) embedded in a polymeric matrix (uronic acids, arabinose, xylose, galactose, rhamnose, fucose, mannose, and glucose). Cyanobacteria cell walls are thicker (10–700 nm) and made up of N-acetylglucosamine and N-acetylmuramic acid covered by a membrane of proteins, lipids, and carotenoids [6]. 

Microalgae production yields 4–15 tons/ha/year in contrast to plant crops, such as wheat, pulse legumes, and soybean, which yield 1.1 tons/ha/year, 1–2 tons/ha/year, and 0.6–1.2 tons/ha/year, respectively [7]. The global market for microalgae was estimated to be about USD 1 billion in 2022 with an annual growth rate of 5.4% from 2023 to 2032 [8]. In Canada, whole algal protein from microalgae *Chlorella protothecoides* strain S106 with a high protein content (>60%) has been approved as an alternative source of protein [9]. 

**Figure 1:**
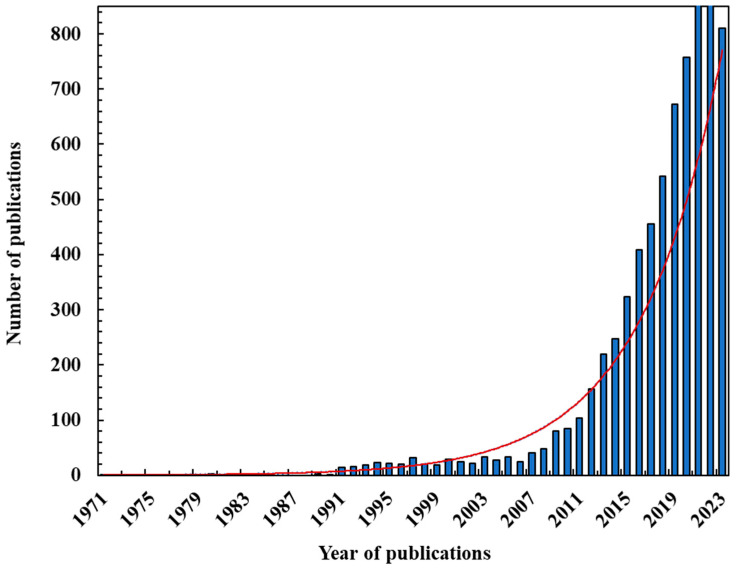
Scientific publications from 1971 to 2023 matching the keyword “microalgae proteins” from Web of Science (https://www.webofscience.com/) accessed on 13 October 2023.

Some microalgae such as *Chlorella* spp., *Arthrospira* spp., and *Euglena* spp. contain significantly larger amounts of protein (50–70%) compared to soy (37%), milk (26%), meat (43%), and yeast (39%). Microalgae also possess a high nutritional quality and low antigenicity [10]. Furthermore, microalgae farming offers promising solutions for mitigating the detrimental effect of population growth, as they utilize anthropic emissions, such as carbon dioxide and ammonium, as a source of nutrients and synthesize value-added macromolecules, such as carbohydrates, lipids, and proteins. Microalgae can adapt to different types of environments, such as water, soil, and climate, compared to conventional crops. Moreover, various microalgae species are considered GRAS (generally recognized as safe) and, hence, can be used for formulating human foods. These microalgae include *Arthrospira platensis*, *Chlamydomonas reinhardtii*, *Auxenochlorella protothecoides*, *Chlorella vulgaris*, *Dunaliella bardawil*, and *Euglena gracilis* [1,11,12]. All these advantages clearly explain the reasons why in the last century the interest in the use of proteins from microalgae has been explored thoroughly as demonstrated by the exponentially growing number of research publications (Figure 1). 

The present review aims to provide a detailed discussion of studies on microalgae, highlighting the advances, opportunities, and challenges related to the potential of using microalgal proteins as sustainable ingredients in novel food formulations. In the first part, the significance of microalgae proteins in human nutrition has been addressed, followed by a discussion of the extraction techniques applied to obtain microalgal proteins with defined physicochemical and functional properties.

## 2. Microalgae for Human Consumption

Microalgae have been incorporated into the human diet for centuries. Chinese used cyanobacteria of the genus Nostoc as food over 2000 years ago. One subsequent utilization of microalgae as food was reported in Mexico around 1524 when Spanish conquistadores discovered that Aztecs made cakes from algae [13]. To date, over 30,000 microalgae species have been identified from over 200,000 to 800,000 existing species [7]. However, only ~10 species are produced for commercial purposes, according to Krishna Koyande et al. [14] and Sousa et al. [15]. Unfortunately, several cases of toxicity and allergenicity have been reported in the literature in common edible microalgae, such as *A. platensis* and *C. vulgaris*. For instance, Petrus et al. [16] reported the first case of anaphylaxis caused by a *Spirulina* dietary supplement in a 14-year-old teenager. Bianco et al. [17] noted the presence of sequence homologs to crustacean food allergens in *Chlorella* and *Spirulina* spp. Particularly, a common allergen, the C-phycocyanin beta subunit was identified in Spirulina. Rzymski et al. [18] also reported the presence of considerable amounts of toxic heavy metals, such as Al or Pb. 

To ensure safety, a deeper knowledge of microalgal biomass toxicity profiles is needed to establish the various species useful for human consumption. Becker [13] suggested different steps for the adoption of microalgal biomass in the human diet: (i) the proximate chemical composition should be provided; (ii) biogenic (phycotoxins and nucleic acids) and non-biogenic (heavy metals and residues from harvesting and processing) toxic compounds should be determined; (iii) protein quality should be assessed; (iv) microbiological control should be performed; and (v) toxicologic and safety tests should be performed. 

In the food industry, microalgae offer a wide range of uses from functional foods to dietary supplements. Their components can also be used as natural dyes. Microalgae are commercially found in different forms, i.e., tablets, capsules, or liquids. Their use has also been reported for food fortification in pasta, candies, ice cream, and beverages. They are also recognized for the extraction of bioactive ingredients, such as β-carotene and phycocyanin [19].

There are over twelve classes of microalgae, of which the four most studied in the literature are: Cyanophyta (blue-green algae), Bacillariophyta (diatoms), Chlorophyta (green algae), and Chrysophyta (golden algae). However, large scale production has been implemented only for a few microalgae, such as Chlorophyceae (*Chlorella* sp. and *Scenedesmus obliquus*) and the cyanobacteria *Athrospira* sp. [20,21].

*Arthrospira platensis* or *Spirulina* (formerly *Spirulina platensis*) is the most widespread microalgae used in the food industry for the production of proteins. It contains a high amount of protein (67.5%), bioactive compounds (50 mg/100 g), polyunsaturated fatty acids (7%), carbohydrates (22%), and minerals. Three other commonly used strains include *Chlorella*, *Dunaliella* salina, and *Aphanizomenon* flos-aquae [19]. *Spirulina* and *Chlorella* are consumed in over 20 countries and are highly appreciated for their high-quality amino acid profiles. *Spirulina* contains about 51% to 71% essential (threonine, valine, methionine, leucine, phenylalanine, tryptophan, and lysine) and non-essential amino acids (glutamic acid, aspartic acid, arginine, alanine, proline, and serine). On the other side, all essential amino acids are present in *Chlorella* (isoleucine, leucine, lysine, methionine, phenylalanine, threonine, tryptophan, valine, and histidine) together with non-essential amino acids such as tyrosine, cystine, aspartic acid, serine, glutamic acid, proline, glycine, alanine, and arginine. Both *Spirulina* and *Chlorella* have reported a similar ratio between essential and non-essential amino acids. Moreover, they have gained increased interest for their strong potential in the production of bioactive compounds (vitamins and carotenoids), for the formulation of functional foods, and for the management of chronic diseases [3]. For instance, *Spirulina* has demonstrated the ability to lower low-density lipoprotein cholesterol and triglyceride levels, reduce blood pressure, and regulate blood sugar levels [7].

## 3. Microalgae Protein Contents and Distribution across Species

**Figure 2:**
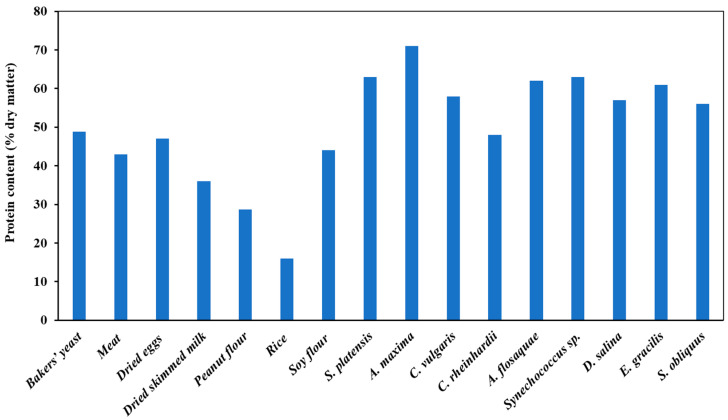
Comparative graph representing the protein content of conventional animal-, fungal-, and plant-based foods and different microalgae species. Adapted from [13,27,28,29,30].

Microalgae are generally made up of carbohydrates (12–30%), lipids (4–20%), and proteins (30–70%) depending on the species (Figure 2). For instance, *Spirulina* sp. contains 50–70% protein, while *Chlorella* contains 50–60%. The use of microalgae as a significant source of dietary protein represents an opportunity to sustainably produce high-quality protein foods. Microalgae also contain other important nutrients such as vitamins A, B1, B2, B6, B12, C, E, and minerals, such as potassium, iron, magnesium, calcium, and iodine [13,22]. Nonetheless, the lack of sufficient food safety data represents a critical limitation. Allergens, hazardous compounds, and contaminants produced during microalgae processing are not comprehensively documented. Yet, safety studies conducted on some microalgae led to conclusive outcomes [23]. For instance, in their study on the safety of Whole Algalin Protein (WAP) derived from dried milled *Chlorella protothecoides*, Szabo et al. [24] reported a high tolerance for WAP with no mutagenicity in rats and no allergenicity in humans.

Different factors regulate the biochemical composition of microalgae, namely the species, culture conditions, growth phase, and physiological conditions [25]. As shown in Figure 2, the protein content of several microalgae is substantially higher than that of common food sources of proteins. For instance, *Arthrospira platensis* contains about 65% protein, which is significantly higher compared to the protein content of conventional sources, such as dried eggs (47%), dried skimmed milk (36%), peanut flour (28.7%), dried meat (40%), soy flour (44%). Depending on the species, the nutritional quality of microalgae protein may also vary. Some microalgae proteins were found to be comparable in quality to conventional proteins. For instance, the amino acid profiles of five different microalgae species (i.e., *Chlorella vulgaris*, *Haematococcus pluvialis*, *Spirulina maxima*, *Diacronema vlkianum*, and *Isochrysis galbana*) were reported to be similar to those of reference food products, such as egg and soybean. In detail, the following essential amino acids were detected: isoleucine, leucine, lysine, methionine, valine, phenylalanine, threonine, tryptophan, and histidine [26].

## 4. Extraction Techniques for Microalgae Proteins

Cultivation and harvesting are crucial steps that determine the quality and safety of microalgae and their derived products. Traditionally, microalgae are cultivated in open raceway pond farms. Nowadays, innovative approaches encourage the use of closed-loop systems, such as photobioreactors. Additionally, freshwater or saline water is being replaced by wastewater as a nutrient source to reduce potential environmental impact [31]. However, it is critical to consider the ability of certain algae to grow in various types of contaminated wastewater. It has been shown that the composition of wastewater has a significant impact on the development of microalgae, the rate of pollutant clearance, and the creation of various intracellular compounds (carbohydrate, protein, and lipid). Moreover, physical factors such as light and nutrients such as nitrogen, sulfur, phosphorus, etc. are critical for microalgae growth. For instance, the protein concentration in *Chlorella vulgaris* increased by over 200% when grown in nitrogen-rich media whereas lipids and polysaccharides were mostly produced when cultured in low-nitrogen media [32]. Biomass harvesting is typically performed as soon as the stationary phase is reached [33]. 

There are various harvesting technologies for microalgae. Flocculation, flotation, filtration, and centrifugation are the most commonly used methods. Centrifugation is the most commonly employed technology for industrial microalgae harvesting due to its simplicity and non-specificity towards species, but it has high costs for energy consumption. In filtration technology, solid and liquid materials are separated by a membrane with pores that allow microalgae culture to pass through while retaining cells. Filtration demonstrated superior performance in harvesting microalgae with the advantage of serving as a preconcentration step before centrifugation. Flotation and flocculation are the preferred methods because of the formation of large aggregates allowing for easy separation of cells by sedimentation [1]. Flotation is the process of attaching bubbles to suspended solid particles, which are brought to the liquid surface due to their low density, thus resulting in separated solid particles. For microalgae with low surface hydrophobicity, flotation efficiency is improved by introducing a collector such as a surfactant or oil emulsion. Together with flotation, flocculation is also considered a low-cost harvesting method. Efficient flocculations are performed in two stages, either by chemical flocculation, bioflocculation, or the use of magnetic nanoparticles [33].

**Figure 3:**
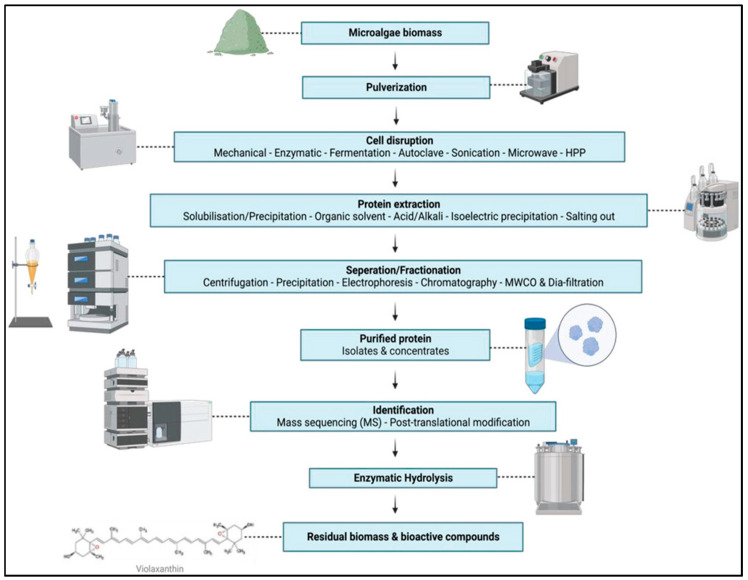
Steps involved in the production of proteins and bioactive compounds from microalgae [4].

Once harvested, different steps are required to process the whole cell into protein-rich ingredients, including cell disruption, protein solubilization, fractionation, purification, and concentration [6]. Figure 3 shows a schematic diagram representing all the steps involved in the process.

The first step includes cell disruption for the release of the intracellular components, such as lipids, proteins, and carbohydrates, into a liquid medium. Cell disruption improves the extraction yields of cellular components because it exposes cellular components to solvents. Cell wall disruption is a critical step due to the rigidity of cell walls in various strains. The resistance of the cell wall is related to the cell wall structure, which, in turn, is controlled by the molecular composition and physicochemical interactions. Many successful methods employed to improve cell disruption are classified into two main categories: mechanical methods (ultrasound, bead milling, high-pressure homogenization, pulse electric field) and non-mechanical methods (enzymatic, acid/base, ionic liquids) [14,22,34]. The mechanical methods appear to be very effective; for instance, high-pressure homogenization was reported to disrupt up to 99.99% of *Chlorella protothecoides* cells [35]. 

To improve cell disruption efficiency, different methods can be combined. For instance, alkaline treatment and ultrasonication were more efficient in disrupting microalgae cells than the individual treatments [36]. Moreover, a protein yield of up to 80% was reported after combining the alkaline and enzymatic extraction of microalgae meal [37]. Nevertheless, the main challenge is the selection of suitable methods that do not compromise the nutritional quality and physicochemical properties of protein extracts due to denaturation or chemical modifications. For solvent extraction, the choice of the solvent and its complete elimination are crucial to avoid safety issues. The reduced efficiency of mechanical extraction methods resides in the lack of specificity in the obtained protein fraction [33]. 

Nowadays, milder and more environmentally friendly processes, such as a pulsed electric field and ultrasound treatments, are emerging as alternatives. For instance, a pulsed electric field was reported to enhance protein yield without affecting functional properties [38]. At the end of this step, a crude extract is obtained consisting of a mixture of cellular components (lipids, proteins, polysaccharides, etc.). Thereafter, further steps are required to obtain the concentrated or purified protein ingredients. Subsequently, a pre-treatment, i.e., solubilization, can be performed. This is generally achieved by modifying the pH to achieve the maximum protein solubility. In this manner, the concentration of soluble proteins increases. This process is followed by protein fractionation, which consists of separating proteins by centrifugation based on their solubility. Two protein fractions are then obtained, i.e., the soluble and insoluble fractions. Proteins from the soluble fraction have been more investigated and utilized than the insoluble fraction due to their desirable functional property (solubility, emulsifying capacity, foaming capacity, and stability) [39,40,41,42]. However, the less functional proteins from the insoluble fractions are also utilized as such or even processed further by hydrolysis to improve their functional properties [35,43]. 

Furthermore, microalgae proteins can be separated from other cell components by additional purification steps. There are two different approaches depending on the extent of purity needed, i.e., refinement and purification. Highly concentrated products require complex extraction processes, thus increasing microalgae production cost, labor, and energy. Different products, from crude proteins to highly concentrated proteins, can be obtained from microalgal biomass. Specifically, these products are purified proteins, proteins isolates, protein concentrates, defatted low-protein meals, and defatted high-protein meals [1,6,44]. Some studies suggest that lipids should be removed before protein extraction. Lipid removal before protein extraction improves the protein yield but may be detrimental to protein quality and functionality. The application of high temperature and organic solvents can induce protein denaturation and aggregation, leading to poor functionality. Therefore, the resulting proteins are less valuable and mostly oriented toward animal feed purposes [6,37]. 

Microalgae protein purification includes different techniques, such as pH shifting [45], three-phase partitioning [46], aqueous two-phase extraction [47], or ultrafiltration [39]. Proteins are later retrieved by precipitation, chromatography, dialysis, and centrifugation [46]. The most common technique used for microalgae protein purification is the isoelectric precipitation method. This process consists of shifting the pH to or near the isoelectric point where solubility is close to zero. Consequently, only proteins precipitating at the same isoelectric point are extracted. Other cell components as well as the proteins with different isoelectric points remain in the supernatant. Hence, this could be considered a selective technique [35]. Filtration is a moderate method based on the physical separation of proteins based on their molecular weight and polarity. Ultrafiltration and diafiltration have been used to concentrate proteins from microalgae. Filtration is solvent-free and energy-efficient. However, this technique uses costly membrane filters, which are exposed to fouling or clogging [39,48]. 

Selective purification of protein from crude extract segregates proteins to obtain protein isolates based on common characteristics. A few examples are precipitation by isoelectric point and ammonium salts, organic solvents, or ionic liquids. The precipitate is later resolubilized and purified by dialysis, ultrafiltration, or membrane filtration. Although it excludes several proteins, precipitation by isoelectric point is mostly used to produce protein-rich isolates [41,44,49,50,51].

## 5. Amino Acid Composition and Digestibility of Microalgae Proteins

Microalgae contain all 20 amino acids and varying levels of indispensable amino acids [19]. The amino acid profile of many microalgae proteins is similar to that of common food sources, such as soybean [52]. The amino acid profile of *Nannochloropsis gaditana* was reported to be similar to the FAO/WHO/UN reference profile [53]. Typically, microalgae are rich in aspartate and glutamate (8–12% total amino acids), whereas the proportions of cysteine, methionine, tryptophan, and histidine can be limited [54,55]. Environmental conditions affect the amino acid composition of microalgae. For example, Xie et al. [56] reported that the essential amino acid index of *Euglena gracilis* differed depending on the nitrogen source, with ammonium sulfate performing better than yeast extract and monosodium glutamate. Nonetheless, Sui et al. [57] demonstrated that the essential amino acid contents of *Dunaliella salina* in the stationary phase were similar and exceeded the FAO/WHO recommended amounts for human nutrition, irrespective of light exposure, i.e., 24 h light or 12 h/12 h light/dark cycles. These results demonstrate the need to optimize the cultivation conditions of individual microalgae to achieve specific nutritional qualities. 

Microalgae proteins of many species may have reduced digestibility compared to conventional sources of food proteins due to the entrapment of some proteins in cell walls. Therefore, microalgae possessing thick cell walls, such as *Chlorella vulgaris*, have lower digestibility than those having thinner cell walls, such as *Spirulina platensis* and *Aphanizomenon flosaquae*. This phenomenon is controlled by the amount of cellulose and other polysaccharides contained in the cell wall, which influences protein extractability and the accessibility of digestive proteases [10]. Proteins are also found inside the cell and in organelles. Intercellular proteins can only interact with digestive enzymes for hydrolysis if the microalgae cells are disrupted. Hence, effective cell disruption improves the protein digestibility of microalgae biomass [10,58]. 

One of the reference methods for assessing digestibility is the protein-digestibility-corrected amino acid score (PDCAAS). This method is based on the amino acid requirements of humans and the ability to digest proteins [59]. Cell disruption and purification influence PDCAAS values. For instance, microalgae protein concentrates and isolates have been reported to have higher PDCAAS values than whole cells. After mechanical cell disruption, the PDCAAS of *Chlorella vulgaris*, *Chlorella sorokiniana*, and *Acutodesmus obliquus* increased from 0.63, 0.64, and 0.29 to 0.77, 0.81, and 0.46, respectively [60,61]. Studies also reported that antinutritional factors found in microalgae, such as phenolic compounds or polysaccharides, could reduce protein digestibility. The oxidation of phenolic compounds and their complexation with protein molecules resulted in insoluble complexes that were resistant to proteolytic enzymes, leading to lower protein digestibility [62]. 

## 6. Physicochemical and Functional Properties of Microalgae Proteins

Several studies have reported the salient physicochemical and functional properties of microalgae proteins, namely solubility, gelation, emulsifying and foaming properties, water-holding capacity, and oil-holding capacity, in isolation or when present within the biomass matrix. These properties are crucial in determining the potential of the microalgae proteins for use in food structuring and product development.

### 6.1. Solubility and Isoelectric Point

Protein solubility can be expressed as the ratio of protein concentration dissolved in aqueous solution relative to the total protein concentration. Protein solubility is controlled by the balance between protein–water interactions and protein–protein interactions. After the centrifugation of aqueous protein solutions, proteins are fractionated into soluble (supernatant) and insoluble (pellet) fractions. Protein solubility is a relevant feature since it controls other functional properties, such as gelation, foaming, and emulsifying capacity. It also affects food quality in terms of viscosity, turbidity, and sedimentation [63,64,65,66]. 

Over the years, several studies have focused on soluble protein fractions with little emphasis on the insoluble fractions. Soluble protein fractions are less pigmented and protein-rich. The functional properties of insoluble protein fractions are quite insubstantial. Owing to their poor dispersibility in water, they are generally used as inert fillers. Nevertheless, recent research strategies tend to optimize protein extraction methods by reducing the yield of inactive fillers. For this purpose, research tends to promote protein extraction in less drastic conditions. The mild modification of the functional properties is highly encouraged. Grossmann et al. [35] promoted the use of less-refined procedures involving cell disruption, protein fractionation, and lyophilization while skipping the purification step.

The soluble fraction of microalgae can be used for food fortification [50,67,68]. Grossmann et al. [50] reported that *Chlorella protothecoides* soluble proteins are solubilized over a broad range of pH (2–12). Generally, since the majority of food proteins are barely soluble in acidic pH, their use in acidic food formulation can be quite challenging. Therefore, microalgae proteins could be a good fit for food formulations over a wide pH range. Protein solubility differs among microalgae. This difference is controlled by the extraction methods, protein isolate concentration, ionic strength, and the type of raw material [69]. Specifically, protein solubility depends on intrinsic (molecular weight, amino acid composition, etc.) and extrinsic factors (pH, ionic strength, etc.). For instance, a study of the protein profile of water-soluble and water-insoluble fractions of *Chlorella protothecoides* showed that the former is mainly composed of hydrophilic polar amino acids [50].

Regarding the relationship between solubility and pH, different studies reported similarities with the behavior of conventional food proteins. Microalgae proteins showed low solubility at pH < 5 and solubility increased from neutral to alkaline pH. For instance, *Tetraselmis* sp. proteins were found to be completely soluble at and above pH 5.5, whereas ionic strength had no impact on solubility [40]. These results aligned with subsequent findings on *Nannochloropsis talian* proteins [45]. Moreover, water-soluble proteins from *Chlorella protothecoides* exhibited a high solubility at pH 2–12 ranging from 84.3% to 100% [50]. The authors concluded that solubility was also affected by glycosylation and hydrophilic amino acid contents. Glycoproteins with their high negative charges improved the solvent–protein interaction. However, ionic strength did not significantly influence microalgae protein solubility. 

**Figure 4:**
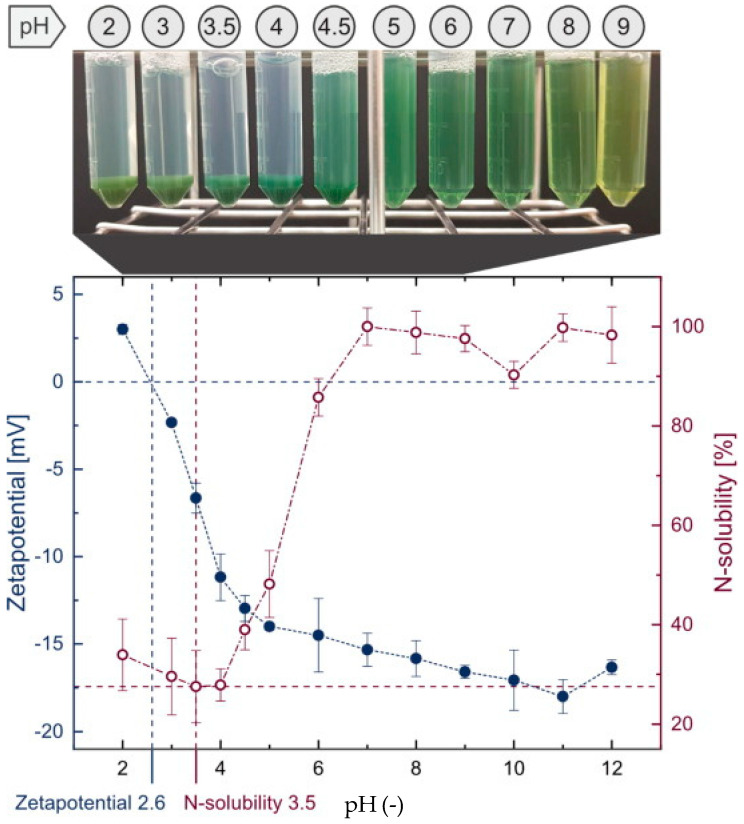
Detection of the isoelectric point of the soluble extract of *Arthrospira platensis* [71].

The solubility of microalgae proteins is low at the isoelectric point when isolated by isoelectric precipitation, where the induced protein–protein interactions cause protein aggregation and precipitation. At other pH values, the microalgae proteins acquire a net positive or negative charge at their surface, favoring protein solubility in water [70]. A recent study on *Arthrospira platensis* identified the isoelectric point of the soluble protein extract to be pH ≤ 4, with zero zeta-potential at pH 2.6 and the least nitrogen solubility at pH 3.5 (Figure 4) [71].

Furthermore, the functional properties of microalgae proteins could be impaired by the presence of high amounts of insoluble proteins. However, the acid hydrolysis of the proteins increased the solubility and interfacial activity, thus improving emulsification and foaming capacities [43,67]. Similarly, enzymatic hydrolysis of *Nannochloropsis gaditana* proteins using papain improved the solubility rate [53]. This occurred because hidden hydrophobic bonds were exposed and the molecular size decreased. Nitrogen solubility is an indication of the protein aggregation index. High nitrogen solubility indicates a poor protein aggregation index. This condition results in high emulsification, foaming, and gelation. Nitrogen solubility is highly influenced by the presence and thickness of the cell wall in the microalgae biomass [52]. The solubilities of many microalgae such as *Spirulina* sp. and *Arthospira platensis* were found to be similar to those of conventional food proteins at neutral pH. This indicates the possibility of using microalgae proteins as sustainable ingredients in food product formulations [69,72].

### 6.2. Water- and Oil-Holding Capacities

Water-holding capacity (WHC) denotes the ability of protein molecules to retain water molecules in their structure. This characteristic is controlled by the presence of polar amino acid residues and the scarcity of water-soluble proteins. Oil-holding capacity (OHC) is the ability of proteins to bind with fat. This feature is particularly important for the mouthfeel characteristics and flavor of food [66].

Several studies have reported a high WHC and OHC for microalgae proteins, suggesting their important role in the formulation of food products with good sensory characteristics. The water-holding capacity of microalgae proteins correlates with pH. According to [73], the WHC of *A. platensis* isolates showed the maximum value (428.8 g of water/100 g of API) at pH 10, whereas there was no significant difference at pH 3 and pH 7. The WHC and OHC of *A. platensis* were respectively lower and higher than those of commercial soy protein isolates.

In a recent study, the WHC of *Haematococcus pluvialis* protein (4.06 g/g) was comparable to that of yellow pea and green lentil protein concentrate but higher than the WHC of red lentil, desi chickpea, and kabuli chickpea protein concentrate [66]. An OHC of 3.29 g/g was reported. This value was comparable to cottonseed protein isolates and chickpea protein isolates, but lower than yellow pea, green lentil, red lentil, desi chickpea, and kabuli chickpea protein concentrates obtained by isoelectric precipitation. 

### 6.3. Gelation

Gelation is an important functional property in several types of food. It is defined as the formation of a three-dimensional gel structure by a network of crosslinked polymers entrapped within a liquid phase. Gels can resist flow and are mechanically rigid over a defined time frame [74]. 

Heat-induced gelation is one of the most prominent processes for food gel production. It involves several events including protein unfolding, denaturation, aggregation, and gelation [75]. Initial reports of gelation mechanisms evaluated on *Arthrospira platensis* protein isolates revealed the minimum critical gelling concentration at pH 7 to be 1.5% in 0.1 M Tris buffer and 2.5% in the same buffer with 0.02 M CaCl_2_ [75]. The result was comparable to later findings where the minimum gelation concentration of microalgae protein was 2.5% (*w*/*v*) at pH 6.5 [34]. Moreover, talian thermo-irreversible gel was formed at temperatures above 60 °C. Below 60 °C, a reversible unfolding of proteins was observed. However, increasing the temperature up to 90 °C for 1 h triggered the aggregation of the protein molecules, resulting in increased elasticity, which was further enhanced upon cooling. The authors indicated that the unfolding exposed hydrophobic residues that were previously hidden in the proteins, which subsequently induced protein aggregation. Therefore, hydrophobic interactions played an important role in the molecular association, initial aggregation, and stability of the protein gels. Hydrogen bonds played a complementary role by improving rigidity. Moreover, the intermolecular sulfhydryl and disulfide bonds control the elasticity of the gel. Time and temperature were retained critical factors for gelation and elasticity between 40 °C and 80 °C. The effect of ionic strength on gelation was also reported; at a low salt concentration (0.004 M CaCl_2_), high elastic gels were obtained at neutral and alkaline pH during the heating stage. Upon an increase in salt concentration (0.02 M calcium chloride), elasticity decreased due to the neutralization effect. Further cooling reduced rigidity at pH of 9 owing to the increase in solubility as revealed by the viscometric measurements. It is worth noting that the presence of protein–pigment complexes greatly promotes the behavior during denaturation and gelation. This observation was rebutted by Shkolnikov Lozober et al. [34], who discovered that phycobiliproteins, the main pigmented protein in *A. platensis*, were not involved in gelation. 

The same observation was reported for *A. platensis* protein isolates, where gelation was controlled by hydrophobic interactions, hydrogen bonds, and intermolecular disulfide bonds [73]. However, the minimal gelation concentration of *A. platensis* isolates in distilled water was higher at 12% (*w*/*w*). Probably the presence of CaCl_2_ contributed to promoting stability by preventing dissociation. Gelation can also be influenced by other processes such as high pressure. Shkolnikov Lozober et al. [34] demonstrated that the gelling capacity of *A. platensis* protein concentrate significantly increased after high-pressure homogenization at 100 and 50 MPa due to the increase in protein hydrophobicity. A stiffer weak gel was obtained. Moreover, the increase in ionic strength reduced the storage modulus of the protein gel. The optimum pH was found to be 6.5; lowering the pH decreased the protein solubility, thus hindering gelation.

In their study, Suarez Garcia et al. [64] investigated the gelation behavior of soluble *Tetraselmis suecica* protein extracts in comparison to whey protein isolates. A heating step (from 25 °C to 90 °C) resulted in protein unfolding and the deformation of a film-like structure caused by further covalent interactions. Gel rigidity increased during the cooling phase (from 90 °C to 25 °C) due to the formation of non-covalent bonds, including hydrogen bonds and hydrophobic interactions. Moreover, microalgae possessed better gelling characteristics than whey protein isolates at 10% protein content. The authors assumed that the other cell components found in the extract (lipids, polysaccharides, and ash) played a part in the rigidity of the gel structure, whereas the small globular proteins found in whey protein isolates did not promote the formation of a stable network.

**Figure 5:**
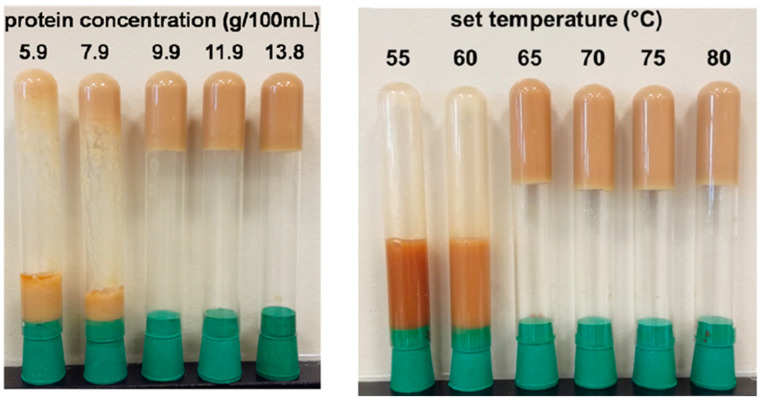
Gelation of water-soluble proteins extracted from *C. sorokiniana* at different protein concentrations (A = Tset = 80 °C for 10 min), and different Tset (B = protein concentration 9.9 g/100 mL) taken from [64].

Grossmann et al. [64] also studied the gelation of protein extract from *Chlorella sorokiniana* (Figure 5). The minimum heat-induced gelling conditions were 9.9 g/100 mL at 80 °C for 10 min. Gelation occurred from 61 °C but 65 °C was enough to obtain a non-pourable gel. This value was comparable to that of whey, pea, and soy proteins. However, the gel was not rigid, and its consistency was similar to stirred yogurt. Temperature exerted a positive effect on gel rigidity, whereas a high ionic strength and variation in pH (pH different from 5.6) affected gelation negatively in terms of elasticity and firmness. The gelling capacity was hindered by hydrolysis, as similarly observed by Shkolnikov Lozober et al. [34] who showed that *A. platensis* concentrate could not form a gel after hydrolysis with pepsin.

Another study on the use of whole microalgae cells as an inert filler in pea/κ-carrageenan/starch mixed gel systems revealed that the addition of *Spirulina* and *Haematococcus* influenced the gel structure, but the gel setting conditions remained constant [76,77]. When compared to control gels, *Haematococcus* gel was highly structured, whereas *Spirulina* gel showed lower values for viscoelastic functions. This drawback was counteracted by reducing the heating/cooling rates.

### 6.4. Emulsifying Properties

Due to their amphiphilic property, proteins are used for the stabilization of emulsions. Emulsions are a mixture of two immiscible liquids. The heterogeneous system formed is made up of a dispersed phase and a continuous phase. The system can be stabilized by using surface-active agents, such as proteins. Proteins can lower the interfacial tension at the oil–water interface, thus facilitating the diffusion of the dispersed phase into the continuous phase. Emulsifying capacity is defined as the oil quantity that can be used to form a stable emulsion [71]. 

Microalgae proteins have been proven to possess good emulsifying properties. For instance, a less-refined lyophilized crude water-soluble extract (WSE) of *Chlorella protothecoides* was capable of stabilizing oil-in-water (o/w) emulsions for up to 7 days, better than the effects of whey proteins [78]. Moreover, emulsions were stable in high-salt conditions (up to 500 mM NaCl) and over a broad range of pH (2–9), making these proteins suitable for different types of food formulations. Similar conclusions were reported by Ebert et al. [68] for water-soluble protein extracts from *Chlorella sorokiniana* and *Phaeodactylum tricornutum*.

The emulsifying capacity of microalgae proteins is correlated to solubility, pH, and protein purification techniques. For instance, in the presence of soluble protein isolates (ASPI) from *Tetraselmis* sp., emulsions at low protein concentrations were stable at a pH of 5–7 [41]. In general, the emulsifying capacity is reduced around the protein isoelectric point where solubility is at its lowest. Above and below the pI, the emulsification capacity increases [73]. *Haematococcus pluvialis* proteins possessed better emulsifying capacities when extracted at neutral pH than those obtained at pH 5.7 [79]. Moreover, the best emulsifying capacity of *Chlorella vulgaris* proteins was obtained at pH 7 [80]. 

The range of pH at which the emulsifying capacity is optimal determines the use of microalgae proteins in food product formulations. Proteins stabilizing emulsions in the acidic range are convenient for acidified emulsions such as beverages, whereas microalgae protein emulsions that are stable at neutral or basic pH would be suitable for neutral and alkaline preparations, respectively. Some microalgae proteins cover a broad range of pH and, therefore, can be used for the formulation of alkaline, neutral, and acidified foods [50].

Some microalgae have demonstrated higher emulsification properties than conventional food proteins or other commercial ingredients. For instance, the emulsification stability of spray-dried *Arthrospira platensis* was higher as compared to egg protein [81]. Similarly, the emulsification capacities of *Porphyridium cruentum* and *Phaeodactylum tricornutum* were higher than that of soy protein [52], whereas the soluble protein extracts from *Chlorella vulgaris* [80] and *Haematococcus pluvialis* [79] were comparable to soy protein or sodium caseinate. Furthermore, the emulsifying capacities of *Arthrospira maxima*, *Nannochloropsis gaditana*, and *Tetraselmis impellucida* protein extracts were similar to those of dairy, legumes, and eggs [82].

Microalgae protein products also stabilize emulsions in different forms, from crude extracts to highly purified forms [49,82]. However, purification improves the emulsification properties of microalgae proteins because of the reduced interference by other components found in crude extracts. For instance, certain lipids and polysaccharides tend to reduce and enhance the emulsification properties, respectively. Also, high protein concentration increases emulsion stability [50,68,71]. Furthermore, the influence of each component of ruptured algal cells on the emulsion properties has been reported [83]. Various components of microalgae play a major role in stabilizing the emulsions, namely cell debris, lipids, and water-soluble fractions. Ruptured cell debris trigger Pickering emulsions as they attach to the droplets surface. Lipids are better surfactants than proteins. However, proteins play a key role in stabilizing emulsions due to the formation of a strong interfacial film. Furthermore, different protein recovery procedures have resulted in different emulsifying capacities. Proteins from tangential ultrafiltration permeate yielded better emulsifying and solubility properties than proteins derived from isoelectric precipitation [80]. This could be due to the occurrence of a high concentration of native and functional proteins in the sample obtained by ultrafiltration.

Böcker et al. [71] also investigated the effect of microalgae protein purification on their emulsifying mechanism and efficiency in o/w emulsion. Proteins were extracted from *A. platensis* biomass and purified leading to a crude extract, soluble extract, protein isolate, and diafiltrated protein isolate. The results showed higher emulsifying properties in crude and soluble extracts than in the microalgae biomass. Purification further increased emulsifying properties, and smaller droplets were observed in the emulsion. The soluble fraction of microalgae proteins is generally utilized in dispersed systems, such as emulsions [41,68,80]. Due to its low water solubility, the insoluble fraction of microalgae proteins shows poor emulsion capacity. One way to improve this parameter is thermal and acid hydrolysis. 

Acid hydrolyzes protein-rich particles into small aggregates and shorter peptide chains, thus increasing solubility. Both the insoluble protein fraction of *Chlorella protothecoides* and its acid hydrolysate were found to stabilize o/w emulsions for 14 days [43]. However, acid hydrolysis improved interfacial activities due to the participation of protein aggregates and protein-aggregate-hydrolyzed peptide interfaces. Protein aggregates favored droplet flocculation, and mixed protein-aggregate-hydrolyzed peptide interfaces hindered coalescence. Moreover, flocculation occurred for all the samples, and droplet size was inversely proportional to the protein concentration and storage time. Emulsion droplets were also stable at higher protein concentrations. Nonetheless, conflicting results were previously reported by Medina et al. [53]. In this study, protein hydrolysates from *Nannochloropsis gaditana* prepared using papain had poor emulsifying capacities. This discrepancy might stem from the difference in the degree of hydrolysis. A high degree of hydrolysis, typically obtainable with acid hydrolysis, implies the generation of smaller peptides and the emergence of several polar groups resulting in an increase in solubility and a reduction in oil absorption capacity. 

### 6.5. Foaming Properties

Foaming properties find their importance in the production of food products, such as whipped cream, meringue, and mousse. Foams are made up of air and water phases and are evaluated based on foaming capacity and foam stability. Foaming capacity is the quantity of interfacial area formed during foaming whereas foam stability is the time required for the foam to keep the same bubble size. Proteins play an important role in improving foaming capacity and foam stability. They are adsorbed at the water–air interface, thus reducing surface tension. This creates viscoelastic interfacial layers that maintain the foam structure and prevent the occurrence of coalescence and drainage. 

Factors controlling the foaming properties of proteins include the source, preparation, extraction and processing methods, composition, solubility, concentration, pH, temperature, and the presence of salts (ionic strength), carbohydrates, and lipids [72]. Studies performed by Benelhadj et al. [73] and Devi and Venkataraman [84] reported that the foaming capacity and foam stability of *Arthrospira platensis* protein isolates was strongly influenced by pH and solubility. Minimum foaming capacity was noted around the isoelectric point where solubility was the lowest, and the highest value occurred at about pH 10. As the net charge of the proteins rises, hydrophobic interaction decreases, thus increasing protein flexibility. Flexible protein molecules can easily diffuse through the air–water interface and improve foam formation. Regarding ionic strength, the authors showed that low salt concentrations increased protein solubility, thus improving foam capacity, and vice versa. Similar outcomes were reported for depigmented *Haematococcus pluvialis* protein isolates [66]. Higher foam stability at alkaline pH was strongly associated with the high surface activity of the proteins. Accordingly, the foaming stability of *Tetraselmis* sp. soluble protein isolates increased with ionic strength (10 mM and 200 mM) but decreased around the isoelectric pH as it was related to protein solubility [42]. 

Furthermore, foaming capacity increases with protein concentration or purification. Buchmann et al. [38] found that foam prepared with crude *Arthrospira platensis* protein-containing powder was less stable than that prepared using *Arthrospira platensis* protein isolates. This conclusion aligned with previous results that reported a higher foaming capacity of *Spirulina* protein concentrate than *Spirulina* flour [84]. Moreover, foaming properties are affected by hydrolysis. Insoluble *Chorella protothecoides* microalgae proteins hydrolyzed at 65 °C and 85 °C were reported to have higher surface activity than untreated samples [67]. Hydrolysis temperature was a critical factor as the best foamability was obtained at higher temperatures; the foam obtained from hydrolysates at 85 °C had a higher volume, smaller bubble diameter, and higher stability. 

Some microalgal proteins have higher foaming stability than conventional food proteins. For example, the soluble algae protein isolate from *Tetraselmis* sp. was reported to form foams that were more stable at pH 5–7 than whey protein isolate and egg white albumin [42]. The foam stability was thought to be due to the presence of proteins alone rather than the contribution of protein–polysaccharide complexes. Similarly, *Haematococcus pluvialis* foaming capacity was higher than that of barley, mung bean protein, chickpea protein isolates, and lupin [66].

## 7. Food Formulated with Microalgae Proteins

Food products formulated with proteins from microalgae currently available in the market are made from whole-cell proteins, protein concentrates, isolates, hydrolysates, and bioactive peptides. Concentrates, isolates, and hydrolysates are categorized based on the degree of purification, which determines their protein content. Whole cells are protein-dense cellular structures containing 40–50% protein, whereas an extraction step and further fractionation are required to obtain protein concentrates, isolates, hydrolysates, and bioactive peptides. These products contain about 60–95% protein [85].

Whole cells are the most popular form in which microalgae are consumed. In this form, they are functionally stable with a weak ability to aggregate and denature owing to their cell wall that prevents pH changes. Furthermore, the presence of significant amounts of value-added products, such as secondary metabolites, offers several advantages for health promotion. These secondary metabolites can be pigments and vitamins. They are utilized as dietary supplements [86]. 

Muñoz-Tebar et al. [87] used whole cells and the ruptured form of *Nannochloropsis salina* in rennet gels and curd to formulate novel dairy products. The whole-cell addition resulted in a better product as the network structure remained undisturbed. However, the appearance of large aggregates destroyed the casein microstructure. A notable advantage of this strategy is the cocktail of beneficial biomolecules that the whole cells can impart to food compared to purified proteins. In addition, functional protein-rich biscuits enriched with omega-3 PUFA such as docosapentaenoic acid, eicosapentaenoic acid were formulated using *Isochrysis galbana*. The addition of the protein-rich microalgae biomass (35%) improved the water absorption capacity, which resulted in firmer and more compact biscuits.

The use of microalgae proteins as whole cells is limited by various factors. Structural and organoleptic properties of formulated foods are altered due to the occurrence of a strong color or pigment, a strong fishy taste and smell, and an increase in firmness. Furthermore, the presence of a rigid cell wall negatively affects the digestibility and the bioavailability of proteins and amino acids [10,43]. However, the issue of pigment-rich microalgae protein extraction has been approached by many researchers. For instance, Grossmann et al. [35] successfully produced less-refined microalgae protein powder extracts with a reduced pigment content and a protein content similar to skim milk powder by combining high-pressure homogenization, fractionation, and lyophilization. 

Table 1, Table 2, Table 3 and Table 4 list scientific studies in which products containing microalgae proteins have been formulated. To encourage downstream cost savings, the use of whole cells is promoted rather than purified proteins. The majority of the products are not commercially available yet, as more in-depth knowledge is required to scale up production and processing. However, some commercially available products in different countries contain microalgae proteins. Lafarga [88] reported the main microalgae-containing products, which are presented in the form of capsules, tablets, and dried products. However, a growing trend of commercially innovative products containing microalgae proteins is gradually entering the global market. While some products utilize only the pigments contained in microalgae, others exploit the nutritional, physicochemical, functional, and sensory properties of microalgae proteins.

*Spirulina* has already been used as an additive in different dairy products such as yogurt, cheese, fermented milk, and vegan kefir [89,90,91,92,93]. Beyond the increase in the protein level, the results also showed that microalgae promoted the growth of lactic acid bacteria and the improvement in the nutritional value of final products. Similar results were also obtained in the case of cheese analog formulated with *Chlorella vulgaris* biomass [94]. 

**Figure 6:**
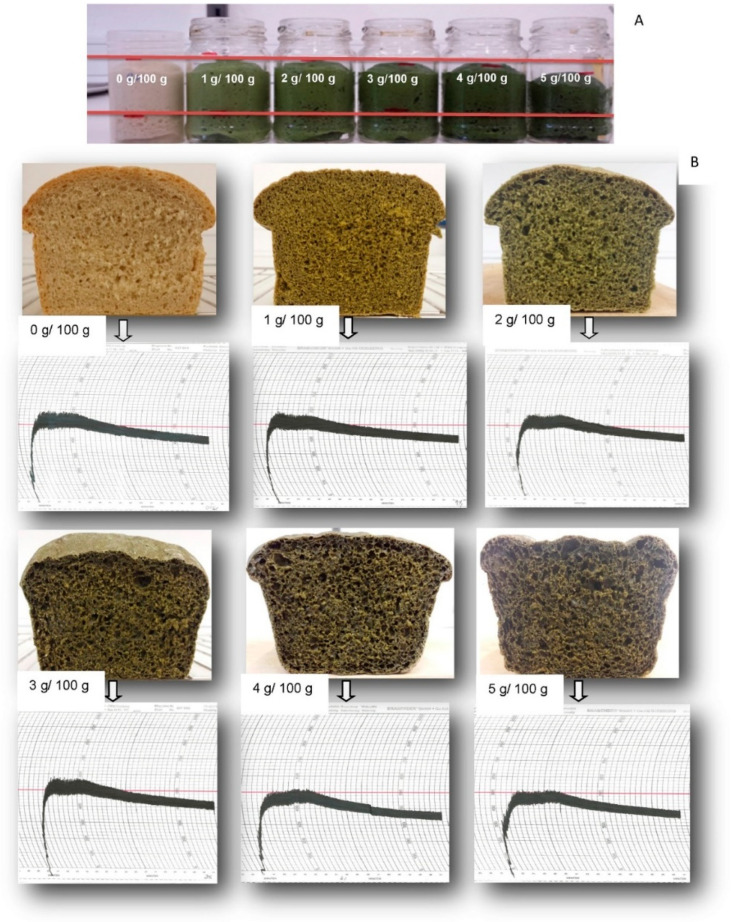
(**A**) Change in volume of bread dough prepared with different concentrations of *Chlorella vulgaris*, after 1 h of fermentation at 37 °C. (**B**) Farinograph analysis of control bread (without *C. vulgaris*) and bread produced with 1, 2, 3, 4, and 5 g *C. vulgaris*/100 g WF (**B**) taken from [102].

Considering bakery products (Table 1), wheat crackers fortified with 2% and 6% microalgae (*Arthrospira platensis*, *Chlorella vulgaris*, *Tetraselmis suecica*, and *Phaeodactylum tricornutum*) had a significantly higher protein content (13.2–14.3%) [95]. Thus, the products could be claimed as a “source of protein”. Additionally, other value-added attributes were observed in the crackers, such as high antioxidant activity. Adding 6% *A. platensis* increased the protein digestibility from 75% to 83%. However, crackers fortified with *C. vulgaris* resulted in a lower digestibility (42%) due to the thick cell wall with high cellulose content. In contrast, higher microalgae content compromised the sensory attributes of the crackers. Similar results were previously reported for wheat cookies enriched with *A. platensis* and *C. vulgaris* [96] and Iranian traditional cookies fortified with *S. platensis* biomass [97]. Based on the results, microalgae proteins are suitable for incorporation into cookie dough when their flavor is pleasant, water absorption capacity is moderate, and protein efficiency is considerable, with slight or no alteration in the dough structure [98]. In contrast, when *Chlorella vulgaris* biomass was added to traditional butter cookies, the high protein content of the microalgae strengthened the dough system, thus increasing firmness [99]. Furthermore, bread enriched with microalgae has been studied [100,101]. For example, the addition of up to 3 g *C. vulgaris* biomass per 100 g of wheat flour improved the viscoelasticity properties of wheat bread by creating a firmer gluten network [102]. Water-holding capacity was also enhanced owing to the additional proteins from the microalgae while the kinetics of yeast fermentation was not affected. However, increasing the amount of *C. vulgaris* reduced the strength and elasticity but improved the extensibility, thus making the dough suitable for biscuits. This de-structuring effect was caused by the disorganization of the gluten network (Figure 6). 

Additionally, crostini, Italian leavened products prepared using sourdough, were enriched with *Arthrospira platensis* [103]. Enriched crostini showed a high protein content with high digestibility (~85%) a microalgae concentration of 6% and 10%. Recently, 3D printing technology has also been employed to produce microalgae-rich products. In 3D printed cookies, the addition of *Arthrospira platensis* and *Chlorella vulgaris* resulted in improved printability and higher mechanical resistance during printing [104]. The authors showed that microalgae biomass are promising ingredients for use as food inks. Changes in eating habits due to veganism and lactose intolerance have led to a search for healthier alternatives (Table 2). Probiotic lactose-free foods and beverages using microalgae have been successfully formulated. For instance, *Arthrospira platensis* (*Spirulina*) was proposed to be a suitable substrate for the production of probiotic-based products [105]. In a subsequent study, *Arthrospira platensis* biomass was added to a soybean drink or in water, as a substrate for lactic acid fermentation by the probiotic bacterium *Lactiplantibacillus plantarum*. The fermented products obtained were rich in highly digestible proteins [106]. Furthermore, some studies have explored the use of fermentation to ameliorate the aromatic profile of substrate and produce probiotic food enriched with lactic acid bacteria. The inclusion of microalgae to plain and probiotic fermented milk counteracted the probiotic reduction issue caused during processing [107]. In another study, *Arthrospira platensis* and *Chlorella vulgaris* increased protein content and improved the viability of probiotics in yogurt at the end of fermentation and during storage [108]. Similar results were reported in subsequent studies [89,109]. Pasta is another food item that shows interesting characteristic features when formulated with microalgae (Table 3) [110]. For example, the addition of microalgae biomass (*Chlorella vulgaris* and *Spirulina maxima*) in fresh semolina spaghetti resulted in the production of protein-rich pasta with improved firmness [111]. One of the interesting features of incorporating microalgae in pasta was the appealing green and orange colors imparted by the microalgae. Cooking did not alter the attributes of the pasta. In their study on the incorporation of *Spirulina* in pasta, [62] noticed an improvement in the nutritional properties of pasta in terms of protein content, phenolic compounds and antioxidant activity. Despite the high protein content, the protein digestibility was reduced, probably due to microalgal antinutritional factors. Conversely, the incorporation of *Chlorella sorokiniana* in pasta resulted in an increase in in vitro digestibility as the microalgae concentration increased [112].

Microalgae have also been utilized for the enrichment of gluten-free products. Gluten-free pasta enriched with *Spirulina* was successfully formulated using rice flour and Psyllium gel [113]. Apart from the high protein content, the gluten-free pasta obtained had high antioxidant activity owing to the presence of phenolic compounds, chlorophylls, and carotenoids from *Spirulina*. The results were in agreement with the studies on gluten-free bread [114] and gluten-free manioc muffins and cakes [115]. Cassava products enriched with microalgae allowed the formulation of inexpensive gluten-free food products with an excellent nutritional profile. Successful outcomes were obtained with cassava doughnuts [116], cassava cakes [117], desserts [118], and noodles [119].

Microalgae have also been used as a structuring ingredient in food (Table 4) [89,107,108,109]. One of the strategies is to utilize whole microalgae cells, thus combining the structuring functionalities with the health-promoting properties of microalgae [120]. For instance, *Arthrospira platensis* improved the rheological and mechanical properties of soy protein isolate hydrogel by enhancing the rigidity and compactness of soy protein isolate structure [121]. The use of *Phaeodactylum tricornutum* in food products was also proposed due to its emulsifying and thickening effects [122]. Earlier, *Spirulina* and *Diacronema* microalgal biomass contribution were monitored in vegetable gelled desserts prepared using 4% pea protein isolate, 0.15% κ-carrageenan, and 2.5% starch. Even though pigments affected the sensory quality of the gel, they imparted thermal stability to the microalgae. Firmer gels were obtained using *Diacronema*. The poor firmness of *Spirulina* gels was attributed to the thermodynamic incompatibility or competitive interaction between pea protein and microalgal protein [110].

Extruded products have also been explored for the utilization of microalgae [100,101,102,103,104]. Some successful examples include formulated protein-rich maize snacks enriched with *Spirulina* [123,124]. Lucas et al. [124] determined the optimal extrusion conditions of maize snacks containing *Spirulina* sp. LEB 18. The *Spirulina* concentration, feed moisture, and temperature of the final zone of the extrusion were reported as key factors affecting the product quality. The optimum conditions were 2.6% *Spirulina*, 16.2% feed moisture, and 143 °C. A higher feed moisture increased product hardness and compactness, thus distorting the expansion of the extruded snacks. The final product in these conditions had a protein content of 11.3%.

Overall, existing studies on the use of microalgae as food ingredients for the formulation of high-protein foods present tremendous opportunities to generate several types of food items, from baked goods to dairy products, with enhanced physicochemical and nutritional properties.

**Table 1:** Bakery products containing microalgae proteins.

Product	Microalgae	Observations	References
Traditional butter cookies	*Chlorella vulgaris*	Increased firmness	[99]
		High protein content of *C. vulgaris* reinforced the dough system.	
		More than 1% *C. vulgaris* altered the cookies color due to the expression of a pronounced green color	
Biscuit	*Isochrysis galbana*	Improved texture properties	[125]
		Color and texture stability	
		High content of polyunsaturated fatty acids	
Manioc (cassava) based bakery products	*A. platensis*	Good texture, expansion coefficient, centesimal composition, and sensory acceptance	[115]
		Added inverted sugar hindered the occurrence of green color	
Cassava cake	*Spirulina platensis*	High protein, vitamins,	[117]
		essential fatty acids, and minerals	
		Good consumer acceptance	
Cassava doughnuts	*Spirulina platensis*	High in protein, minerals, fiber, and lipids	[116]
		Addition of 5.41% *S. platensis* plus 10.0% inverted sugar resulted in good sensory scores	
Bread wheat pasta	*Spirulina platensis*	High protein content	[62]
		High phenolic compound content and antioxidant activity	
		Surface heterogenicity with 20% *Spirulina*	
		Decrease protein digestibility	
Iranian traditional cookies	*Spirulina platensis*	High iron, protein, and γ-linolenic acid content	[97]
		High sensory scores obtained with 1–1.5% *S. platensis*	
Extruded snacks	*Spirulina platensis*	Addition of *Spirulina* sp. LEB 18, temperature in the last zone of the extruder, and feed moisture are critical factors for the snack quality	[124]
		Increasing *Spirulina* concentration improved protein content and compactness	
Wheat cookies	*Arthrospira platensis*, *Chlorella vulgaris Allma*, *Tetraselmis suecica*	Higher protein content obtained with *A. platensis* and *C. vulgaris*	[96]
	*Phaeodactylum tricornutum*	Better texture obtained with *A. platensis*	
		Higher antioxidant capacity and total phenolic content	
Wheat flour bread	*Chlorella vulgaris*	Negative impact on bread quality when more than 3% *C. vulgaris* was added.	[102]
		Gluten network reinforcement (≤3%).	
		High in bioactive compounds	
		Increase in water-holding capacity	
Wheat crackers	*Arthrospira platensis*, *Chlorella vulgaris Allma*, *Tetraselmis suecica*, *Phaeodactylum tricornutum*	Higher protein content in cookies obtained with *A. platensis* and *C. vulgaris*. Proteins have the claim “source of protein”	[95]
		High-antioxidant crackers obtained with *A. platensis*, *T. suecica*, and *P. tricornutum*	
		High sensory scores with *A. platensis*	
		Low sensory scores with *T. suecica* and *P. tricornutum*	
Breads and crackers	*Tetraselmis* and *Nannochloropsis*	Optimum results obtained with a microalgae concentration of 2.5% for baked crackers and 1.0 or 2.0% for breads	[126]
		Darker and greener color	
		Improved nutritional value with high protein and antioxidant content	
Sourdough “crostini”	*Spirulina platensis*	“Source of protein” claim with 6% and 10% biomass	[127]
		High in protein and antioxidants	
		Lower in vitro dry matter and protein digestibility than control, but still above 85%	
Gluten-free bread	*Nannochloropsis gaditana* and *Chlamydomonas* sp.	More protein, lipids, and ash than the control bread	[114]
		Microalgae had a structuring effect on the gluten-free bread texture: more adhesive and firm structure	
		Highest sensory score obtained for 3% *N. gaditana* L2 bread	
Gluten free bread	*Chlorella sorokiniana*	Improved protein content from 67 mg g^−1^ to 85 mg g^−1^	[128]
Gluten free bread	*Tetraselmis chuii*	Optimum concentration: 4% *Tetraselmis chuii*	[129]
Wheat bread	*Tetraselmis chuii*	Protein-rich, high-quality bread	[130]
		Treatment of *T. chuii* with ethanol lowered the unpleasant color and improved dough rheology	
		Improved protein and bioactivity over control	
Wheat tortillas	*Nannochloropsis* sp. and *Tetraselmis* sp.	High protein and fat content	[131]
		High antioxidant activity and phenolic content, especially in flour enriched with 3% *Nannochloropsis* sp.	
Bread	*Spirulina platensis*	2–6% *Spirulina* led to more nutritional bread	[101]
Indonesian milk pie (Pie Susu) made up with modified cassava flour	*Spirulina platensis*	Good consumer acceptance obtained with 0.5% *Spirulina*	[118]
Bread	*Spirulina platensis*	Greener color with increasing concentration from 1.5 to 2.5% addition. The 2.5% concentration samples were well accepted by consumers, emphasizing the salty flavor as a pleasant feature	[132]
Muffin	*Chlorella vulgaris*	Effect on microstructure and texture with 1.5% microalgae	[133]
Low-saturated-fat bread	*Chlorella vulgaris*	High protein and low saturated fat. High water content affecting bread’s sensory scores	[134]

**Table 2:** Dairy products containing microalgae proteins.

Product	Microalgae	Observations	References
Fermented acidophilus–bifidus–thermophilus (ABT) milks	*Spirulina platensis*	Positive effect on the survival of ABT starter bacteria	[93]
		Enrichment in bioactive molecules	
Yogurt	*Spirulina platensis*	Improved viability of lactic acid bacteria	[90]
Yogurt	*Chlorella vulgaris* and *Arthrospira platensis*	Improved viability of yogurt cultures	[108]
Yogurt	*Spirulina platensis*	High protein, fat, and iron content	[109]
		Curd strength proportional to microalgae concentration	
		Sensory score of 0.3% for yogurt was comparable to the control	
		Higher viability of yogurt culture	
Enriched feta cheese containing *Lactobacillus acidophilus* and *Mentha longifolia* L.	*Spirulina platensis*	Stimulatory effect on the growth and viability of probiotic bacteria	[91]
		Improved protein and iron content	
Bread	*Isochrysis galbana*, *Tetraselmis suecica*, *Scenedesmus almeriensis* and *Nannochloropsis gaditana*	Addition of microalga had no significant effect on hardness, chewiness, or resilience over the control sample	[100]
Ayran (western Asian yogurt-based beverage)	*S. platensis*	*S. platensis* improved the growth of probiotics	[89]
Ice cream	*Spirulina platensis*	35% to 53% more proteins in the enriched ice cream	[135]
		High acceptability index (70%)	
3D printed cookies	*Arthrospira platensis* and *Chlorella vulgaris*	High mechanical resistance	[104]
		High elasticity	
		Improves the printability	
		High stability and resistance to baking of 3D structures	
Chocolate milk	*Spirulina platensis*	High protein content and reduced total lipids	[26]
		High antioxidant activity and phenolic content	[136]
Yogurt	*Spirulina platensis*	High protein content upon the addition of phycocyanin from *Spirulina*	[137]
Renneted dairy gels and curd	*Nannochloropsis salina*	Rennet action undisturbed	[87]
		Whole cells did not change the gel structure	
		Ruptured cells destroyed casein microstructures	
Cheese	*Spirulina platensis* and *Chlorella vulgaris*	Significant increase in antioxidant activity, iron, and total phenolic content	[138]

**Table 3:** Pasta containing microalgae proteins.

Product	Microalgae	Observations	References
Fresh spaghetti	*Chlorella vulgaris* and *Spirulina*	Color stable after cooking	[111]
	*Maxima*	High firmness in raw pasta	
		High sensory acceptance than control pasta	
Semolina spaghetti	*Isochrysis galbana* and *Diacronema*	High protein content	[139]
	*Vlkianum*	High resistance to the thermal treatment	
		High omega-3 fatty acid content	
Microalgae as a substrate for lactic acid fermentation plantarum	*Spirulina platensis*	High antioxidant content	[105]
		*A. platensis* is a suitable substrate for *L. plantarum* growth	
Vegetal soybean drink	*Spirulina platensis*	*S. platensis* biomass suitable substrate for LAB8014 growth	[106]
		High protein content	
		Better digestibility	
Pasta	*Spirulina*	Microencapsulation of *Spirulina* contributes to antioxidant preservation	[140]
Gluten-free pasta	*Spirulina platensis*	Products 2% *Arthrospira platensis* had consumer acceptance	[113]
		No significant change in pasta texture caused by the addition of microalgae	
		Higher protein and antioxidant content	
Mocaf noodles	*Spirulina platensis*	More chewy, dense, and not easily broken noodle	[119]
Pasta	*Chlorella sorokiniana*	Appearance of fish flavor when more than 5% *C. sorokiniana* was added	[112]
		High in protein and PUFA	
		High antioxidant content	
Whole wheat Pasta	*Himanthalia elongata* and *Spirulina*	Increase in fat, protein, ash, total amino acid contents, and antioxidant activity	[141]

**Table 4:** Other products derived from microalgae.

Product	Microalgae	Observations	References
Vegetable-based gelled desserts (pea protein isolate)	*Spirulina maxima* and *Diacronema* *Vlkianum*	Microalgae cells were resistant to thermal treatments *D. vlkianum* conferred more firmness than *S. maxima*	[110]
Gels prepared from pea protein, κ-carrageenan and starch	*Spirulina* and *Haematococcus*	More structured gels obtained upon temperature increase (70–90 °C)	[26]
Cheese analogue	*Chlorella vulgaris*	Improved protein, carbohydrate, and fiber contents Product with more firmness and strong network	[94]
Enriched dehydrated soup	*Spirulina platensis*	High in protein, fiber, lipids, antioxidant activity, and total phenolic content	[142]
		Occurrence of a characteristic green color and herb flavor	
		Good consumer acceptability and intent to purchase	
Broccoli soup	*Spirulina* sp., *Chlorella* sp., or	Higher concentration of bioaccessible polyphenols	[143]
	*Tetraselmis* sp.	Higher consumer acceptance (70%)	
Vegan kefir	*Spirulina platensis*	High lactobacilli and lactococci count	[92]
		Increased total phenolic content of kefir	
		Decreased pH	
Soy protein isolate hydrogel (SPI)	*Spirulina platensis*	Improved rigidity and compactness of SPI hydrogel	[121]
Plant-based meat alternatives	*Spirulina* and *Chlorella*	Higher gumminess and chewiness	[144]
Vegetable creams	*Arthrospira platensis* (*Spirulina*), *Chlorella vulgaris*, *Tetraselmis chui*, or *Nannochloropsis oceanica*	Improved protein content and amino acid nutritional profile. No significant differences in protein digestibility	[145]
Vegan oil-in-water emulsion	*Spirulina platensis*	Interesting rheological parameters compared with a more traditional protein source such as chickpea	[146]

## 8. Challenges and Future Prospects

Proteins from microalgae are a relatively new entrant in the market of sustainable non-conventional compounds. Owing to the increasing consumer interest in health promoting and clean-label products, the adoption of microalgae proteins in the human diet presents a promising future. Microalgae are rich in proteins with a good amino profile comparable to conventional food proteins. Functional properties such as gelling capacity, emulsifying properties, and foaming properties make them promising candidates for various applications in the food industry as non-animal-based protein substitutes or new products to fill the protein gap. Microalgae may contribute to the formulation of novel products fulfilling emerging consumer food habits, such as veganism and vegetarianism. Moreover, microalgae proteins are appreciated for their health-promoting properties. They can contribute to the reduction in cardiovascular disease risk factors as microalgae-derived biopeptides possess valuable hypertensive and antioxidant properties [147]. When used as whole cells, their health benefits considerably expand to other cell components, such as phenolics, vitamins, or enzymes. Sustainable cultivation is another attractive feature of microalgae. Cultivation technologies such as closed-loop systems require less water and land than methods used to produce traditional protein sources. 

However, various challenges need to be addressed before the wide utilization of microalgae protein becomes effective. One of the biggest issues is consumer awareness. Owing to the recent insertion of microalgae proteins into the market, consumer awareness is still in its early stages. Only a small percentage of the world population is aware of the benefits of microalgae consumption as a protein source. A study conducted in Spain reported that about 85% of the population declared that there was a lack of in-depth information about microalgae [148]. The same observation was made in Italy [149]. Consumer awareness strategies should be implemented through programs that could communicate a positive image of microalgae, especially related to their roles in achieving environmental sustainability, health promotion, and food security.

Moreover, cultural barriers constitute a major limitation to the use of microalgae proteins. The color and typical “marine” taste of microalgae are not generally appreciated by many consumers. Several studies are finding strategies to mask the undesirable flavors either by incorporating microalgae in food formulations, by adding spices [149], or by modifying the aromatic profile through fermentation [150]. However, the sensory issue is far from being mitigated.

To date, the large-scale production of microalgae biomass has not been widely implemented. High production costs and technical challenges related to biomass variability, contamination, and nutrient availability are not well elucidated due to gaps in scientific knowledge. Scaling up may also result in high energy production due to downstream processing. This implies potential environmental issues, such as greenhouse gas emissions and impact on ecosystems. Additionally, this sector suffers from the incomplete or lack of a regulatory framework for product safety and labeling. 

Consequently, research must be intensified to provide insights into the efficient production and safe consumption of microalgae and their protein products [151]. Continued advancements and developments are still required to enhance the efficiency of the processing of microalgae proteins. Moreover, the interactions of microalgae proteins with other constituents of the food matrix are yet to be completely understood for their effective utilization. Future studies should investigate the effect of residual pigments after protein extraction on the digestibility of microalgae proteins. Optimal and critical processing parameters must be determined to improve product quality.

Finally, information on safety would arguably increase the willingness of consumers to buy microalgae-based products. This would have a knock-on effect on microalgae economics and the cost of protein production.

## Data Availability

No new data were created or analyzed in this study. Data sharing is not applicable to this article.

## References

[1] Amorim M.L., Soares J., dos Reis Coimbra J.S., de Oliviera Leite M., Albino L.F.T., Martins M.A. (2021). Microalgae Proteins: Production, Separation, Isolation, Quantification, and Application in Food and Feed. Crit. Rev. Food Sci. Nutr..

[2] Falcon W.P., Naylor R.L., Shankar N.D. (2022). Rethinking Global Food Demand for 2050. Popul. Dev. Rev..

[3] Caporgno M.P., Mathys A. (2018). Trends in Microalgae Incorporation into Innovative Food Products with Potential Health Benefits. Front. Nutr..

[4] Eilam Y., Khattib H., Pintel N., Avni D. (2023). Microalgae—Sustainable Source for Alternative Proteins and Functional Ingredients Promoting Gut and Liver Health. Glob. Chall..

[5] Brasil B.d.S.A.F., de Siqueira F.G., Salum T.F.C., Zanette C.M., Spier M.R. (2017). Microalgae and Cyanobacteria as Enzyme Biofactories. Algal Res..

[6] Grossmann L., Hinrichs J., Weiss J. (2019). Cultivation and Downstream Processing of Microalgae and Cyanobacteria to Generate Protein-Based Technofunctional Food Ingredients. Crit. Rev. Food Sci. Nutr..

[7] Koyande A.K., Chew K.W., Rambabu K., Tao Y., Chu D.T., Show P.L. (2019). Microalgae: A Potential Alternative to Health Supplementation for Humans. Food Sci. Hum. Wellness.

[8] Polaris Market Research Microalgae Market Size, Share Global Analysis Report, 2023–2032. https://www.polarismarketresearch.com/industry-analysis/microalgae-market/toc.

[9] Health Canada. https://www.canada.ca/en/health-canada/services/food-nutrition/genetically-modified-foods-other-novel-foods/approved-products/whole-algal-protein.html.

[10] Soto-Sierra L., Stoykova P., Nikolov Z.L. (2018). Extraction and Fractionation of Microalgae-Based Protein Products. Algal Res..

[11] Torres-Tiji Y., Fields F.J., Mayfield S.P. (2020). Microalgae as a Future Food Source. Biotechnol. Adv..

[12] Baier T., Kros D., Feiner R.C., Lauersen K.J., Müller K.M., Kruse O. (2018). Engineered Fusion Proteins for Efficient Protein Secretion and Purification of a Human Growth Factor from the Green Microalga *Chlamydomonas reinhardtii*. ACS Synth. Biol..

[13] Becker E.W. (2013). Microalgae for Human and Animal Nutrition. Handbook of Microalgal Culture.

[14] Krishna Koyande A., Tanzil V., Murraly Dharan H., Subramaniam M., Robert R.N., Lau P.L., Khoiroh I., Show P.L. (2020). Integration of Osmotic Shock Assisted Liquid Biphasic System for Protein Extraction from Microalgae Chlorella Vulgaris. Biochem. Eng. J..

[15] Sousa I., Gouveia L., Batista A.P., Raymundo A., Bandarra N.M. (2008). Microalgae in Novel Food Products. Food Chemistry Research Developments.

[16] Petrus M., Culerrier R., Campistron M., Barre A., Rougé P. (2010). First Case Report of Anaphylaxis to Spirulin: Identification of Phycocyanin as Responsible Allergen. Allergy.

[17] Bianco M., Ventura G., Calvano C.D., Losito I., Cataldi T.R.I. (2022). A New Paradigm to Search for Allergenic Proteins in Novel Foods by Integrating Proteomics Analysis and in Silico Sequence Homology Prediction: Focus on Spirulina and Chlorella Microalgae. Talanta.

[18] Rzymski P., Budzulak J., Niedzielski P., Klimaszyk P., Proch J., Kozak L., Poniedziałek B. (2019). Essential and Toxic Elements in Commercial Microalgal Food Supplements. J. Appl. Phycol..

[19] Spolaore P., Joannis-Cassan C., Duran E., Isambert A. (2006). Commercial Applications of Microalgae. J. Biosci. Bioeng..

[20] Chen P., Min M., Chen Y., Wang L., Li Y., Chen Q., Wang C., Wan Y., Wang X., Cheng Y. (2009). Review of the Biological and Engineering Aspects of Algae to Fuels Approach. Int. J. Agric. Biol. Eng..

[21] Sheehan J., Dunahay T., Benemann J., Roessler P. (1998). Look Back at the U.S. Department of Energy’s Aquatic Species Program: Biodiesel from Algae.

[22] Timira V., Meki K., Li Z., Lin H., Xu M., Pramod S.N. (2022). A Comprehensive Review on the Application of Novel Disruption Techniques for Proteins Release from Microalgae. Crit. Rev. Food Sci. Nutr..

[23] Fu Y., Chen T., Chen S.H.Y., Liu B., Sun P., Sun H., Chen F. (2021). The Potentials and Challenges of Using Microalgae as an Ingredient to Produce Meat Analogues. Trends Food Sci. Technol..

[24] Szabo N.J., Matulka R.A., Chan T. (2013). Safety Evaluation of Whole Algalin Protein (WAP) from *Chlorella protothecoides*. Food Chem. Toxicol..

[25] Hu Q. (2013). Environmental Effects on Cell Composition. Handbook of Microalgal Culture: Applied Phycology and Biotechnology.

[26] Batista A.P., Gouveia L., Bandarra N.M., Franco J.M., Raymundo A. (2013). Comparison of Microalgal Biomass Profiles as Novel Functional Ingredient for Food Products. Algal Res..

[27] Xing Q., de Wit M., Kyriakopoulou K., Boom R.M., Schutyser M.A.I. (2018). Protein Enrichment of Defatted Soybean Flour by Fine Milling and Electrostatic Separation. Innov. Food Sci. Emerg. Technol..

[28] Hoogenkamp H., Kumagai H., Wanasundara J.P.D. (2017). Rice Protein and Rice Protein Products. Sustainable Protein Sources.

[29] Asen N.D., Badamasi A.T., Gborigo J.T., Aluko R.E., Girgih A.T. (2021). Comparative Evaluation of the Antioxidant Properties of Whole Peanut Flour, Defatted Peanut Protein Meal, and Peanut Protein Concentrate. Front. Sustain. Food Syst..

[30] Takalloo Z., Nikkhah M., Nemati R., Jalilian N., Sajedi R.H. (2020). Autolysis, Plasmolysis and Enzymatic Hydrolysis of Baker’s Yeast (*Saccharomyces cerevisiae*): A Comparative Study. World J. Microbiol. Biotechnol..

[31] Srimongkol P., Sangtanoo P., Songserm P., Watsuntorn W., Karnchanatat A., Shiong Khoo K., Wei Lim J. (2022). Microalgae-Based Wastewater Treatment for Developing Economic and Environmental Sustainability: Current Status and Future Prospects. Front. Bioeng. Biotechnol..

[32] Guccione A., Biondi N., Sampietro G., Rodolfi L., Bassi N., Tredici M.R. (2014). Chlorella for Protein and Biofuels: From Strain Selection to Outdoor Cultivation in a Green Wall Panel Photobioreactor. Biotechnol. Biofuels.

[33] Vandamme D., Foubert I., Muylaert K. (2013). Flocculation as a Low-Cost Method for Harvesting Microalgae for Bulk Biomass Production. Trends Biotechnol..

[34] Shkolnikov Lozober H., Okun Z., Shpigelman A. (2021). The Impact of High-Pressure Homogenization on Thermal Gelation of *Arthrospira platensis* (Spirulina) Protein Concentrate. Innov. Food Sci. Emerg. Technol..

[35] Grossmann L., Ebert S., Hinrichs J., Weiss J. (2018). Production of Protein-Rich Extracts from Disrupted Microalgae Cells: Impact of Solvent Treatment and Lyophilization. Algal Res..

[36] Phong W.N., Show P.L., Le C.F., Tao Y., Chang J.S., Ling T.C. (2018). Improving Cell Disruption Efficiency to Facilitate Protein Release from Microalgae Using Chemical and Mechanical Integrated Method. Biochem. Eng. J..

[37] Sari Y.W., Bruins M.E., Sanders J.P.M. (2013). Enzyme Assisted Protein Extraction from Rapeseed, Soybean, and Microalgae Meals. Ind. Crops Prod..

[38] Buchmann L., Bertsch P., Böcker L., Krähenmann U., Fischer P., Mathys A. (2019). Adsorption Kinetics and Foaming Properties of Soluble Microalgae Fractions at the Air/Water Interface. Food Hydrocoll..

[39] Safi C., Olivieri G., Campos R.P., Engelen-Smit N., Mulder W.J., van den Broek L.A.M., Sijtsma L. (2017). Biorefinery of Microalgal Soluble Proteins by Sequential Processing and Membrane Filtration. Bioresour. Technol..

[40] Schwenzfeier A., Wierenga P.A., Gruppen H. (2011). Isolation and Characterization of Soluble Protein from the Green Microalgae *Tetraselmis* sp.. Bioresour. Technol..

[41] Schwenzfeier A., Helbig A., Wierenga P.A., Gruppen H. (2013). Emulsion Properties of Algae Soluble Protein Isolate from *Tetraselmis* sp.. Food Hydrocoll..

[42] Schwenzfeier A., Lech F., Wierenga P.A., Eppink M.H.M., Gruppen H. (2013). Foam Properties of Algae Soluble Protein Isolate: Effect of PH and Ionic Strength. Food Hydrocoll..

[43] Dai L., Hinrichs J., Weiss J. (2020). Emulsifying Properties of Acid-Hydrolyzed Insoluble Protein Fraction from *Chlorella protothecoides*: Formation and Storage Stability of Emulsions. Food Hydrocoll..

[44] Bertsch P., Böcker L., Mathys A., Fischer P. (2021). Proteins from Microalgae for the Stabilization of Fluid Interfaces, Emulsions, and Foams. Trends Food Sci. Technol..

[45] Cavonius L.R., Albers E., Undeland I. (2015). PH-Shift Processing of *Nannochloropsis oculata* Microalgal Biomass to Obtain a Protein-Enriched Food or Feed Ingredient. Algal Res..

[46] Waghmare A.G., Salve M.K., Leblanc J.G., Arya S.S. (2016). Concentration and Characterization of Microalgae Proteins from *Chlorella pyrenoidosa*. Bioresour. Bioprocess..

[47] Suarez Ruiz C.A., Kwaijtaal J., Peinado O.C., Van Den Berg C., Wijffels R.H., Eppink M.H.M. (2020). Multistep Fractionation of Microalgal Biomolecules Using Selective Aqueous Two-Phase Systems. ACS Sustain. Chem. Eng..

[48] Suarez Garcia E., van Leeuwen J., Safi C., Sijtsma L., Eppink M.H.M., Wijffels R.H., van den Berg C. (2018). Selective and Energy Efficient Extraction of Functional Proteins from Microalgae for Food Applications. Bioresour. Technol..

[49] Caporgno M.P., Haberkorn I., Böcker L., Mathys A. (2019). Cultivation of *Chlorella protothecoides* under Different Growth Modes and Its Utilisation in Oil/Water Emulsions. Bioresour. Technol..

[50] Grossmann L., Hinrichs J., Weiss J. (2019). Solubility and Aggregation Behavior of Protein Fractions from the Heterotrophically Cultivated Microalga *Chlorella protothecoides*. Food Res. Int..

[51] Schwenzfeier A., Wierenga P.A., Eppink M.H.M., Gruppen H. (2014). Effect of Charged Polysaccharides on the Techno-Functional Properties of Fractions Obtained from Algae Soluble Protein Isolate. Food Hydrocoll..

[52] Guil-Guerrero J.L., Navarro-Juárez R., López-Martínez J.C., Campra-Madrid P., Rebolloso-Fuentes M. (2004). Functional Properties of the Biomass of Three Microalgal Species. J. Food Eng..

[53] Medina C., Rubilar M., Shene C., Torres S., Verdugo M. (2015). Protein Fractions with Techno-Functional and Antioxidant Properties from *Nannochloropsis gaditana* Microalgal Biomass. J. Biobased Mater. Bioenergy.

[54] Dunford N.T. (2012). Food and Industrial Bioproducts and Bioprocessing.

[55] Volkman J.K., Brown M.R. (2006). Nutritional Value of Microalgae and Applications. Algal Cultures, Analogues of Blooms and Applications.

[56] Xie W., Li X., Xu H., Chen F., Cheng K.-W., Liu H., Liu B. (2023). Optimization of Heterotrophic Culture Conditions for the Microalgae Euglena Gracilis to Produce Proteins. Mar. Drugs.

[57] Sui Y., Muys M., Vermeir P., D’Adamo S., Vlaeminck S.E. (2019). Light Regime and Growth Phase Affect the Microalgal Production of Protein Quantity and Quality with Dunaliella Salina. Bioresour. Technol..

[58] Bleakley S., Hayes M. (2017). Algal Proteins: Extraction, Application, and Challenges Concerning Production. Foods.

[59] Schaafsma G. (2000). The Protein Digestibility–Corrected Amino Acid Score. J. Nutr..

[60] Wang Y., Tibbetts S.M., Berrue F., McGinn P.J., MacQuarrie S.P., Puttaswamy A., Patelakis S., Schmidt D., Melanson R., MacKenzie S.E. (2020). A Rat Study to Evaluate the Protein Quality of Three Green Microalgal Species and the Impact of Mechanical Cell Wall Disruption. Foods.

[61] Wang Y., Tibbetts S.M., McGinn P.J. (2021). Microalgae as Sources of High-Quality Protein for Human Food and Protein Supplements. Foods.

[62] Rodríguez De Marco E., Steffolani M.E., Martínez C.S., León A.E. (2014). Effects of Spirulina Biomass on the Technological and Nutritional Quality of Bread Wheat Pasta. LWT.

[63] Grossmann L., Ebert S., Hinrichs J., Weiss J. (2018). Effect of Precipitation, Lyophilization, and Organic Solvent Extraction on Preparation of Protein-Rich Powders from the Microalgae *Chlorella protothecoides*. Algal Res..

[64] Grossmann L., Hinrichs J., Goff H.D., Weiss J. (2019). Heat-Induced Gel Formation of a Protein-Rich Extract from the Microalga *Chlorella sorokiniana*. Innov. Food Sci. Emerg. Technol..

[65] Haque M.A., Timilsena Y.P., Adhikari B. (2016). Food Proteins, Structure, and Function. Ref. Modul. Food Sci..

[66] Zhu Y., Zhao X., Zhang X., Liu H. (2019). Extraction, Structural and Functional Properties of *Haematococcus pluvialis* Protein after Pigment Removal. Int. J. Biol. Macromol..

[67] Dai L., Shivananda R., Hinrichs J., Weiss J. (2020). Foaming of Acid-Hydrolyzed Insoluble Microalgae Proteins from *Chlorella protothecoides*. Food Biophys..

[68] Ebert S., Grossmann L., Hinrichs J., Weiss J. (2019). Emulsifying Properties of Water-Soluble Proteins Extracted from the Microalgae *Chlorella sorokiniana* and *Phaeodactylum tricornutum*. Food Funct..

[69] Chen Y., Chen J., Chang C., Chen J., Cao F., Zhao J., Zheng Y., Zhu J. (2019). Physicochemical and Functional Properties of Proteins Extracted from Three Microalgal Species. Food Hydrocoll..

[70] Pelegrine D.H.G., Gasparetto C.A. (2005). Whey Proteins Solubility as Function of Temperature and PH. LWT Food Sci. Technol..

[71] Böcker L., Bertsch P., Wenner D., Teixeira S., Bergfreund J., Eder S., Fischer P., Mathys A. (2021). Effect of *Arthrospira platensis* Microalgae Protein Purification on Emulsification Mechanism and Efficiency. J. Colloid Interface Sci..

[72] Pereira A.M., Lisboa C.R., Costa J.A.V. (2018). High Protein Ingredients of Microalgal Origin: Obtainment and Functional Properties. Innov. Food Sci. Emerg. Technol..

[73] Benelhadj S., Gharsallaoui A., Degraeve P., Attia H., Ghorbel D. (2016). Effect of PH on the Functional Properties of *Arthrospira* (*Spirulina*) *platensis* Protein Isolate. Food Chem..

[74] Dickinson E. (1991). Food Polymers, Gels and Colloids.

[75] Chronakis I.S. (2001). Gelation of Edible Blue-Green Algae Protein Isolate (*Spirulina platensis* Strain Pacifica): Thermal Transitions, Rheological Properties, and Molecular Forces Involved. J. Agric. Food Chem..

[76] Batista A.P., Nunes M.C., Fradinho P., Gouveia L., Sousa I., Raymundo A., Franco J.M. (2012). Novel Foods with Microalgal Ingredients–Effect of Gel Setting Conditions on the Linear Viscoelasticity of *Spirulina* and *Haematococcus* Gels. J. Food Eng..

[77] Batista A.P., Nunes M.C., Raymundo A., Gouveia L., Sousa I., Cordobés F., Guerrero A., Franco J.M. (2011). Microalgae Biomass Interaction in Biopolymer Gelled Systems. Food Hydrocoll..

[78] Grossmann L., Ebert S., Hinrichs J., Weiss J. (2019). Formation and Stability of Emulsions Prepared with a Water-Soluble Extract from the Microalga *Chlorella protothecoides*. J. Agric. Food Chem..

[79] Ba F., Ursu A.V., Laroche C., Djelveh G. (2016). *Haematococcus pluvialis* Soluble Proteins: Extraction, Characterization, Concentration/Fractionation and Emulsifying Properties. Bioresour. Technol..

[80] Ursu A.V., Marcati A., Sayd T., Sante-Lhoutellier V., Djelveh G., Michaud P. (2014). Extraction, Fractionation and Functional Properties of Proteins from the Microalgae *Chlorella vulgaris*. Bioresour. Technol..

[81] Nirmala C., Prakash V., Venkataraman L.V. (1992). Physico-Chemical and Functional Properties of Proteins from Spray Dried Algae (*Spirulina platensis*). Food/Nahrung.

[82] Teuling E., Schrama J.W., Gruppen H., Wierenga P.A. (2019). Characterizing Emulsion Properties of Microalgal and Cyanobacterial Protein Isolates. Algal Res..

[83] Law S.Q.K., Mettu S., Ashokkumar M., Scales P.J., Martin G.J.O. (2018). Emulsifying Properties of Ruptured Microalgae Cells: Barriers to Lipid Extraction or Promising Biosurfactants?. Colloids Surf. B Biointerfaces.

[84] Devi M.A., Venkataraman L.V. (1984). Functional Properties of Protein Products of Mass Cultivated Blue-Green Alga *Spirulina platensia*. J. Food Sci..

[85] Teuling E., Wierenga P.A., Schrama J.W., Gruppen H. (2017). Comparison of Protein Extracts from Various Unicellular Green Sources. J. Agric. Food Chem..

[86] Ferreira de Oliveira A.P., Bragotto A.P.A. (2022). Microalgae-Based Products: Food and Public Health. Future Foods.

[87] Muñoz-Tebar N., Ong L., Gamlath C.J., Yatipanthalawa B.S., Ashokkumar M., Gras S.L., Berruga M.I., Martin G.J.O. (2022). Nutrient Enrichment of Dairy Curd by Incorporation of Whole and Ruptured Microalgal Cells (*Nannochloropsis salina*). Innov. Food Sci. Emerg. Technol..

[88] Lafarga T. (2019). Effect of Microalgal Biomass Incorporation into Foods: Nutritional and Sensorial Attributes of the End Products. Algal Res..

[89] Çelekli A., Alslibi Z.A., Bozkurt H. (2019). Influence of Incorporated Spirulina Platensis on the Growth of Microflora and Physicochemical Properties of Ayran as a Functional Food. Algal Res..

[90] Guldas M., Irkin R. (2010). Influence of Spirulina Platensis Powder on the Microflora of Yoghurt and Acidophilus Milk. Časopis Za Unaprjeđenje Proizv. Prerade Mlijeka.

[91] Mazinani S., Fadaei V., Khosravi-Darani K. (2016). Impact of Spirulina Platensis on Physicochemical Properties and Viability of *Lactobacillus acidophilus* of Probiotic UF Feta Cheese. J. Food Process. Preserv..

[92] Sözeri Atik D., Gürbüz B., Bölük E., Palabıyık İ. (2021). Development of Vegan Kefir Fortified with Spirulina Platensis. Food Biosci..

[93] Varga L., Szigeti J., Kovács R., Földes T., Buti S. (2002). Influence of a *Spirulina platensis* Biomass on the Microflora of Fermented ABT Milks during Storage (R1). J. Dairy Sci..

[94] Mohamed A.G., Abo-El-Khair B.E., Shalaby S.M. (2013). Quality of Novel Healthy Processed Cheese Analogue Enhanced with Marine Microalgae *Chlorella vulgaris* Biomass. World Appl. Sci. J..

[95] Batista A.P., Niccolai A., Bursic I., Sousa I., Raymundo A., Rodolfi L., Biondi N., Tredici M.R. (2019). Microalgae as Functional Ingredients in Savory Food Products: Application to Wheat Crackers. Foods.

[96] Batista A.P., Niccolai A., Fradinho P., Fragoso S., Bursic I., Rodolfi L., Biondi N., Tredici M.R., Sousa I., Raymundo A. (2017). Microalgae Biomass as an Alternative Ingredient in Cookies: Sensory, Physical and Chemical Properties, Antioxidant Activity and in Vitro Digestibility. Algal Res..

[97] Shahbazizadeh S., Khosravi-Darani K., Sohrabvandi S. (2015). Fortification of Iranian Traditional Cookies with *Spirulina platensis*. Annu. Res. Rev. Biol..

[98] Kadam S.U., Prabhasankar P. (2010). Marine Foods as Functional Ingredients in Bakery and Pasta Products. Food Res. Int..

[99] Gouveia L., Batista A.P., Miranda A., Empis J., Raymundo A. (2007). Chlorella Vulgaris Biomass Used as Colouring Source in Traditional Butter Cookies. Innov. Food Sci. Emerg. Technol..

[100] García-Segovia P., Pagán-Moreno M.J., Lara I.F., Martínez-Monzó J. (2017). Effect of Microalgae Incorporation on Physicochemical and Textural Properties in Wheat Bread Formulation. Food Sci. Technol. Int..

[101] Saharan V., Jood S. (2021). Effect of Storage on Spirulina Platensis Powder Supplemented Breads. J. Food Sci. Technol..

[102] Graça C., Fradinho P., Sousa I., Raymundo A. (2018). Impact of Chlorella Vulgaris on the Rheology of Wheat Flour Dough and Bread Texture. LWT.

[103] Niccolai A., Chini Zittelli G., Rodolfi L., Biondi N., Tredici M.R. (2019). Microalgae of Interest as Food Source: Biochemical Composition and Digestibility. Algal Res..

[104] Uribe-Wandurraga Z.N., Igual M., Reino-Moyón J., García-Segovia P., Martínez-Monzó J. (2021). Effect of Microalgae (*Arthrospira platensis* and *Chlorella vulgaris*) Addition on 3D Printed Cookies. Food Biophys..

[105] Niccolai A., Shannon E., Abu-Ghannam N., Biondi N., Rodolfi L., Tredici M.R. (2019). Lactic Acid Fermentation of *Arthrospira platensis* (Spirulina) Biomass for Probiotic-Based Products. J. Appl. Phycol..

[106] Niccolai A., Bažec K., Rodolfi L., Biondi N., Zlatić E., Jamnik P., Tredici M.R. (2020). Lactic Acid Fermentation of *Arthrospira platensis* (Spirulina) in a Vegetal Soybean Drink for Developing New Functional Lactose-Free Beverages. Front. Microbiol..

[107] Parada J.L., De Caire G.Z., De Mulé M.C.Z., De Cano M.M.S. (1998). Lactic Acid Bacteria Growth Promoters from Spirulina Platensis. Int. J. Food Microbiol..

[108] Beheshtipour H., Mortazavian A.M., Haratian P., Khosravi-Darani K. (2012). Effects of Chlorella Vulgaris and *Arthrospira platensis* Addition on Viability of Probiotic Bacteria in Yogurt and Its Biochemical Properties. Eur. Food Res. Technol..

[109] Priyanka M., Kempanna C., Narasimha M. (2013). Quality Characteristics of Yoghurt Enriched with Spirulina Powder. Mysore J. Agric. Sci..

[110] Gouveia L., Batista A.P., Raymundo A., Bandarra N. (2008). *Spirulina maxima* and *Diacronema vlkianum* Microalgae in Vegetable Gelled Desserts. Nutr. Food Sci..

[111] Fradique M., Batista A.P., Nunes M.C., Gouveia L., Bandarra N.M., Raymundo A. (2010). Incorporation of *Chlorella vulgaris* and *Spirulina maxima* Biomass in Pasta Products. Part 1: Preparation and Evaluation. J. Sci. Food Agric..

[112] Bazarnova J., Nilova L., Trukhina E., Bernavskaya M., Smyatskaya Y., Aktar T. (2021). Use of Microalgae Biomass for Fortification of Food Products from Grain. Foods.

[113] Fradinho P., Niccolai A., Soares R., Rodolfi L., Biondi N., Tredici M.R., Sousa I., Raymundo A. (2020). Effect of *Arthrospira platensis* (Spirulina) Incorporation on the Rheological and Bioactive Properties of Gluten-Free Fresh Pasta. Algal Res..

[114] Khemiri S., Khelifi N., Nunes M.C., Ferreira A., Gouveia L., Smaali I., Raymundo A. (2020). Microalgae Biomass as an Additional Ingredient of Gluten-Free Bread: Dough Rheology, Texture Quality and Nutritional Properties. Algal Res..

[115] Danesi E.D.G., Navacchi M.F.P., Takeuchi K.P., Frata M.T., Carvalho J.C.M. (2010). Application of *Spirulina platensis* in Protein Enrichment of Manioc Based Bakery Products. J. Biotechnol..

[116] Rabelo S.F., Lemes A.C., Takeuchi K.P., Frata M.T., de Carvalho J.C.M., Danesi E.D.G. (2013). Development of Cassava Doughnuts Enriched with *Spirulina platensis* Biomass. Braz. J. Food Technol..

[117] Navacchi M.F.P., de Carvalho J.C.M., Takeuchi K.P., Danesi E.D.G. (2012). Development of Cassava Cake Enriched with Its Own Bran and Spirulina Platensis. Acta Sci. Technol..

[118] Ariani R., Ekayani I., Masdarini L. (2021). Processing Mocaf into Pie Susu with the Addition of Super Food “Spirulina”. J. Phys. Conf. Ser..

[119] Al-Baarri A.N., Widayat, Aulia R., Prahasiwi E.K., Mawarid A.A., Pangestika W., Lestari F.P. (2021). The Hardness Analysis of Noodles Made from Modified Cassava Flour, Spirulina (*Spirulina platensis*) and Basil Leaves Extract (*Ocimum sanctum* L.). IOP Conf. Ser. Earth Environ. Sci..

[120] Bernaerts T.M.M., Gheysen L., Foubert I., Hendrickx M.E., Van Loey A.M. (2019). The Potential of Microalgae and Their Biopolymers as Structuring Ingredients in Food: A Review. Biotechnol. Adv..

[121] Wang M., Yin Z., Sun W., Zhong Q., Zhang Y., Zeng M. (2023). Microalgae Play a Structuring Role in Food: Effect of Spirulina Platensis on the Rheological, Gelling Characteristics, and Mechanical Properties of Soy Protein Isolate Hydrogel. Food Hydrocoll..

[122] Castro-Ferreira C., Gomes-Dias J.S., Ferreira-Santos P., Pereira R.N., Vicente A.A., Rocha C.M.R. (2022). *Phaeodactylum tricornutum* Extracts as Structuring Agents for Food Applications: Physicochemical and Functional Properties. Food Hydrocoll..

[123] Joshi S.M.R., Bera M.B., Panesar P.S. (2014). Extrusion Cooking of Maize/Spirulina Mixture: Factors Affecting Expanded Product Characteristics and Sensory Quality. J. Food Process. Preserv..

[124] Lucas B.F., de Morais M.G., Santos T.D., Costa J.A.V. (2017). Effect of *Spirulina* Addition on the Physicochemical and Structural Properties of Extruded Snacks. Food Sci. Technol..

[125] Gouveia L., Coutinho C., Mendonça E., Batista A.P., Sousa I., Bandarra N.M., Raymundo A. (2008). Functional Biscuits with PUFA-Ω3 from Isochrysis Galbana. J. Sci. Food Agric..

[126] Lafarga T., Mayre E., Echeverria G., Viñas I., Villaró S., Acién-Fernández F.G., Castellari M., Aguiló-Aguayo I. (2019). Potential of the Microalgae *Nannochloropsis* and *Tetraselmis* for Being Used as Innovative Ingredients in Baked Goods. LWT.

[127] Niccolai A., Venturi M., Galli V., Pini N., Rodolfi L., Biondi N., D’Ottavio M., Batista A.P., Raymundo A., Granchi L. (2019). Development of New Microalgae-Based Sourdough “Crostini”: Functional Effects of *Arthrospira platensis* (Spirulina) Addition. Sci. Rep..

[128] Diprat A.B., Silveira Thys R.C., Rodrigues E., Rech R. (2020). *Chlorella sorokiniana*: A New Alternative Source of Carotenoids and Proteins for Gluten-Free Bread. LWT.

[129] Nunes M.C., Fernandes I., Vasco I., Sousa I., Raymundo A. (2020). *Tetraselmis chuii* as a Sustainable and Healthy Ingredient to Produce Gluten-Free Bread: Impact on Structure, Colour and Bioactivity. Foods.

[130] Qazi W.M., Ballance S., Uhlen A.K., Kousoulaki K., Haugen J.E., Rieder A. (2021). Protein Enrichment of Wheat Bread with the Marine Green Microalgae *Tetraselmis chuii*–Impact on Dough Rheology and Bread Quality. LWT.

[131] Hernández-López I., Benavente Valdés J.R., Castellari M., Aguiló-Aguayo I., Morillas-España A., Sánchez-Zurano A., Acién-Fernández F.G., Lafarga T. (2021). Utilisation of the Marine Microalgae *Nannochloropsis* sp. and *Tetraselmis* sp. as Innovative Ingredients in the Formulation of Wheat Tortillas. Algal Res..

[132] Hernández-López I., Alamprese C., Cappa C., Prieto-Santiago V., Abadias M., Aguiló-Aguayo I. (2023). Effect of Spirulina in Bread Formulated with Wheat Flours of Different Alveograph Strength. Foods.

[133] Marzec A., Kramarczuk P., Kowalska H., Kowalska J. (2023). Effect of Type of Flour and Microalgae (*Chlorella vulgaris*) on the Rheological, Microstructural, Textural, and Sensory Properties of Vegan Muffins. Appl. Sci..

[134] Pereira T., Costa S., Barroso S., Teixeira P., Mendes S., Gil M.M. (2024). Development and Optimization of High-Protein and Low-Saturated Fat Bread Formulations Enriched with Lupin and Microalgae. LWT.

[135] Tiepo C.B.V., Gottardo F.M., Mortari L.M., Bertol C.D., Reinehr C.O., Colla L.M. (2021). Addition of *Spirulina platensis* in Handmade Ice Cream: Phisicochemical and Sensory Effects Adição de *Spirulina platensis* Em Sorvete Caseiro: Efeitos Físico-Químicos e Sensoriais. Braz. J. Dev..

[136] Batista de Oliveira T.T., Miranda dos Reis I., Bastos de Souza M., da Silva Bispo E., Fonseca Maciel L., Druzian J.I., Lordelo Guimarães Tavares P.P., de Oliveira Cerqueira A., dos Santos Boa Morte E., Abreu Glória M.B. (2021). Microencapsulation of *Spirulina* sp. LEB-18 and Its Incorporation in Chocolate Milk: Properties and Functional Potential. LWT.

[137] Arslan R., Aksay S. (2022). Investigation of Sensorial and Physicochemical Properties of Yoghurt Colored with Phycocyanin of *Spirulina platensis*. J. Food Process. Preserv..

[138] Jalili S., Aryan S., Mousavinezhad S.A., Moeini H., Dehnad D. (2024). Optimizing *Spirulina platensis*, *Chlorella vulgaris* Microalgae and Curcumin Application in Functional Cheese Production and Investigating Its Physicochemical Properties and Sensory Evaluation by RSM. J. Food Meas. Charact..

[139] Fradique M., Batista A.P., Nunes M.C., Gouveia L., Bandarra N.M., Raymundo A. (2013). *Isochrysis galbana* and *Diacronema vlkianum* Biomass Incorporation in Pasta Products as PUFA’s Source. LWT.

[140] Zen C.K., Tiepo C.B.V., da Silva R.V., Reinehr C.O., Gutkoski L.C., Oro T., Colla L.M. (2020). Development of Functional Pasta with Microencapsulated Spirulina: Technological and Sensorial Effects. J. Sci. Food Agric..

[141] Oliveira B.C.C., Machado M., Machado S., Costa A.S.G., Bessada S., Alves R.C., Oliveira M.B.P.P. (2023). Algae Incorporation and Nutritional Improvement: The Case of a Whole-Wheat Pasta. Foods.

[142] Los P.R., Simões D.R.S., Leone R.d.S., Bolanho B.C., Cardoso T., Danesi E.D.G. (2018). Viability of Peach Palm By-Product, *Spirulina Platensis*, and Spinach for the Enrichment of Dehydrated Soup. Pesqui. Agropecu. Bras..

[143] Lafarga T., Acién-Fernández F.G., Castellari M., Villaró S., Bobo G., Aguiló-Aguayo I. (2019). Effect of Microalgae Incorporation on the Physicochemical, Nutritional, and Sensorial Properties of an Innovative Broccoli Soup. LWT.

[144] Bakhsh A., Park J., Baritugo K.A., Kim B., Sil Moon S., Rahman A., Park S. (2023). A Holistic Approach toward Development of Plant-Based Meat Alternatives through Incorporation of Novel Microalgae-Based Ingredients. Front. Nutr..

[145] Prandi B., Boukid F., Van De Walle S., Cutroneo S., Comaposada J., Van Royen G., Sforza S., Tedeschi T., Castellari M. (2023). Protein Quality and Protein Digestibility of Vegetable Creams Reformulated with Microalgae Inclusion. Foods.

[146] Braga A.R.C., Nunes M.C., Raymundo A. (2023). The Experimental Development of Emulsions Enriched and Stabilized by Recovering Matter from *Spirulina biomass*: Valorization of Residue into a Sustainable Protein Source. Molecules.

[147] Ejike C.E.C.C., Collins S.A., Balasuriya N., Swanson A.K., Mason B., Udenigwe C.C. (2017). Prospects of Microalgae Proteins in Producing Peptide-Based Functional Foods for Promoting Cardiovascular Health. Trends Food Sci. Technol..

[148] Lafarga T., Rodríguez-Bermúdez R., Morillas-España A., Villaró S., García-Vaquero M., Morán L., Sánchez-Zurano A., González-López C.V., Acién-Fernández F.G. (2021). Consumer Knowledge and Attitudes towards Microalgae as Food: The Case of Spain. Algal Res..

[149] Lafarga T., Pieroni C., D’imporzano G., Maggioni L., Adani F., Acién G. (2021). Consumer Attitudes towards Microalgae Production and Microalgae-Based Agricultural Products: The Cases of Almería (Spain) and Livorno (Italy). ChemEngineering.

[150] Martelli F., Cirlini M., Lazzi C., Neviani E., Bernini V. (2020). Solid-State Fermentation of *Arthrospira platensis* to Implement New Food Products: Evaluation of Stabilization Treatments and Bacterial Growth on the Volatile Fraction. Foods.

[151] Vázquez-Romero B., Perales J.A., Pereira H., Barbosa M., Ruiz J. (2022). Techno-Economic Assessment of Microalgae Production, Harvesting and Drying for Food, Feed, Cosmetics, and Agriculture. Sci. Total Environ..

